# Carnivores and their prey in Sumatra: Occupancy and activity in human-dominated forests

**DOI:** 10.1371/journal.pone.0265440

**Published:** 2022-03-18

**Authors:** Febri Anggriawan Widodo, Muhammad Ali Imron, Sunarto Sunarto, Anthony J. Giordano

**Affiliations:** 1 World Wide Fund for Nature (WWF) Indonesia, Central Sumatra, Pekanbaru, Indonesia; 2 Wildlife Laboratory, Faculty of Forestry, Universitas Gadjah Mada, Yogyakarta, Indonesia; 3 S.P.E.C.I.E.S. – The Society for the Preservation of Endangered Carnivores and their International Ecological Study, Ventura, California, United States of America; 4 International Development Studies, Faculty of Geoscience, Utrecht University, Utrecht, the Netherlands; 5 Institute for Sustainable Earth and Resources (I-SER), University of Indonesia, Depok, Indonesia; Texas State University, UNITED STATES

## Abstract

Understanding the effect of anthropogenic disturbance, and its interaction with carnivores and their prey, is crucial to support the conservation of threatened carnivores, particularly in rapidly changing landscapes. Based on systematic camera-trap sampling of four protected areas in Riau Province of central Sumatra, we assessed the habitat occupancy and spatiotemporal overlap between people, potential carnivore prey, and four threatened species of medium-sized or large carnivores: Sumatran tigers (*Panthera tigris sumatrae*), Malayan sun bears (*Helarctos malayanus*), dholes (*Cuon alpinus*), and Sunda clouded leopards (*Neofelis diardi*). To assess spatial overlap of target species, we used single-species occupancy models and applied a Species Interaction Factor (SIF) to conditional two-species occupancy models. We also used kernel density estimation (KDE) to assess temporal overlap among these species. Our habitat use models showed that altitude (elevation) strongly influenced the occupancy of all large carnivores and potential prey species. Except for Sunda clouded leopards, the occurrence of large carnivore species was positively related to the spatial co-occurrence of humans (SIF > 1). In addition, we found that sun bears and dholes both exhibited high spatial overlap with tigers, and that sun bears alone exhibited high temporal overlap with people. Our findings contribute to an improved understanding of the contemporary ecology of carnivores and their prey in rapidly changing, southeast Asian landscapes. Such knowledge is important to the conservation and recovery of large carnivores in conservation hotspots that are increasingly dominated by humans across Sumatra, as well as globally.

## Introduction

Sumatra is globally significant for large carnivore conservation and now supports the only extant populations of ’island’ tigers [1, 2]. Efforts to conserve carnivore habitats on Sumatra have been centered around a number of protected areas, including “conservation areas” (*i*.*e*. wildlife sanctuaries, national parks) and “protected forests” (*i*.*e*., designated protected forest areas) [3, 4]. However, human intrusion and habitat degradation continue to occur in and around these protected areas, driven mainly by illegal subsistence and commercial agricultural activities [4, 5]. This impact is further aggravated by the direct poaching of carnivores and their prey, as well as retaliatory killings precipitated by human-carnivore conflict. Both of these activities are substantial factors in the rate of decline of local carnivore populations in Sumatra and globally, as well as the contraction of their geographic ranges [6, 7]. Strong anthropogenic pressures tend to not only eliminate large carnivores, but also have cascading effects on ecosystem function [8]. Overall, approximately 7,540,000 ha of primary forest was lost in Sumatra between 1990–2010; moreover, between 1985 and 2007, the ‘conservation area’ and ‘protection forest’ classes of protected areas have lost 12% and 20% of forest cover, respectively, anthropogenic impacts which are having serious repercussions for suitable carnivore habitats on the island [9, 10].

Environmental conditions, including anthropogenic disturbances, and the presence of other species, can influence species occurrences [11]. Understanding the effects of those environmental conditions, and the presence of other species on sympatric carnivores, is critical to improving conservation management strategies. Such studies are sparse however and require current information regarding species habitat and population dynamics, including the effects of anthropogenic disturbance intensity [8, 12, 13]. Furthermore, it is important that these studies investigate species co-occurrence patterns as a means to examine interspecific interactions in the context of these disturbances [14]. Co-occurrence or co-occupancy analyses offer an effective way to investigate the interaction between species [15]. Our study investigates the relationships among carnivores, potential prey, and humans via spatiotemporal occupancy data. We used systematic camera-trapping surveys to address the challenges related to collecting ecological occurrence information on large forest carnivores, which often present a research challenge due to their elusiveness, low population densities, and dense rainforest vegetation [1, 16, 17]. To account for potential “false absences” of our target species and robustly examine potential competition and the importance of species and habitat covariates, we used two-species occupancy models and incorporated a species interaction factor (SIF) [18]. In addition, we used kernel density estimation (KDE) based on circular-transformed temporal data to investigate the temporal overlap and interaction among people, large carnivores, and potential prey species, [17].

Our study further contributes to contemporary ecological insights into the ecology of carnivores and their prey across a large, anthropogenically-impacted landscape that includes four major protected areas in central Sumatra. Moreover, it fills critical gaps in our general knowledge of these carnivores both overall and for the island. Several prior scientific studies in Sumatra have described various aspects of the ecology of carnivores, including: interspecific interactions among five sympatric cat species [2]; the ecology of meso-predators, including Sunda clouded leopards (*Neofelis diardi*), marbled cats (*Pardofelis marmorata*), golden cats (*Catopuma temminckii*) and leopard cats (*Prionailurus bengalensis*) [19]; the activity patterns of five sympatric cat species [20]; and, temporal overlap between Sumatran tigers (*Panthera tigris sumatrae*) and other native carnivores [21]. Few studies have examined the impact of humans on carnivore ecology and occupancy in Sumatra beyond variation in population densities for tigers [22–24], and Sunda clouded leopards [25] in a limited geographical region. No scientific study has of yet explored the spatial and temporal overlap among people, sympatric carnivores, and their potential prey species, in Sumatra. To this end, we aimed to assess the occupancy and habitat relationships of large carnivorans, including the Sumatran tiger, Malayan sun bear (*Helarctos malayanus*), dhole (*Cuon alpinus*), and Sunda clouded leopard, and their potential prey species, including ungulates such as the southern red muntjac (*Muntiacus muntjac*), bearded pig (*Sus barbatus*), sambar deer (*Rusa unicolor*), Sumatran serow (*Capricornis sumatraensis*), wild boar (*Sus scrofa*) and a smaller prey species group, mouse deer (*Trangulus spp*). Our objective was to do this in the greater context of anthropogenic impacts to understand the role of human presence and different human activities. We also intended to investigate the spatiotemporal overlap exhibited among humans, large carnivores, and their potential prey species, as well as examine the overlap and interactions among tigers as the local apex predator, other ‘subordinate’ large carnivores, and potential prey species.

## Materials and methods

We obtained all relevant permits from The Ministry of Environment and Forestry under its technical agencies: Balai Besar Konservasi Sumber Daya Alam Riau (BBKSDA Riau/Natural Resource Conservation Agency of Riau), and Tesso Nilo National Park (TNNP). In addition, our field teams also received verbal permissions from all adjacent villages to work across our study sites.

### Study area

From 2012 to 2015, we conducted systematic camera-trapping surveys of six different study sites within four different management regimes of protected areas in the southern part of Riau Province, including: Bukit Rimbang Bukit Baling Wildlife Reserve (RBWR or Rimbang Baling for short), Tesso Nilo National Park (TNNP), Bukit Bungkuk Nature Reserve (BBNR), and Bukit Betabuh Protection Forest (BBPF). For each study area, we attempted to select sampling sites that were generally and broadly representative. RBWR (1,410 km^2^) is the largest protected area among all our study sites, and sampling was conducted in three parts of the reserve: the northeast (surveyed in 2012), the northwest (surveyed in 2014), and the south (surveyed in 2015). TNNP (830 km^2^), a national park established in 2004, was sampled in 2013, whereas BBNR, the smallest conservation area surveyed (200 km^2^), was sampled in 2012. BBPF, the only “protected forest”, BBP sampled in 2013.

### Data collection

We deployed paired camera-trap stations originally intended to facilitate the identification of individual tigers (*i*.*e*., taking photos of each flank of an animal), record their prey, and identify other sympatric species, including other carnivores. We superimposed a digital 2x2-km grid over all study sites using ArcGIS [26], and selected every other cell to sample to minimize spatial autocorrelation of data. The interval among camera-trap stations ranged between one and four kilometers to assure that at least three pairs of cameras were present in each tiger’s home range [22]. Cell size was originally chosen as a compromise between the logistics and cost of field access and navigation, and a statistical need to ensure a ‘non-zero probability’ of photographing target species, especially tigers [2]. Camera-traps were left in the field for ~3 months to meet the assumption of a demographically closed population, as the gestation period of tigers is reported to range from 90 to 120 days [22, 25], and installed at a height of ~30 cm above ground level. The sensitivity of camera sensors varied according to the physical context of the station location (*e*.*g*., cameras were set to medium sensitivity under less dense/ more open canopy). We did not install camera traps in tree canopies to record the arboreal activities of species. Camera-traps paired at the same station were deployed at a slight angle away from each other to avoid having to trigger each other. All camera-traps were visited monthly to retrieve data from memory cards and ensure continued camera functionality. We did not use any lures or baits to attract animals, and used several different passive infrared camera-trap models across study areas, including: Bushnell^®^ Natureview and Trophycam models, and Reconyx^®^ HC600 and PC800 models. All data were managed via spreadsheets and compiled into a singular standardized database [22, 27].

For our analyses, we focused on ten different wildlife species as well as humans. These species included all large and medium-sized carnivores (body size: 20.50 to 185.50 kg), (*i*.*e*., tigers, clouded leopards, dholes, sun bears) [2], but also included large- and medium-sized potential prey species like southern red muntjac, bearded pigs, sambar deer, Sumatran serows, wild pigs, and mouse deer [2, 28, 29]. We designated events as independent if either (1) consecutive images were of different species, or (2) and interval of at least 30 minutes occurred between consecutive images of the same species [30]. For each station and overall, we calculated average camera-trapping rates (CTR) for each species by dividing the number of independent “capture” events by camera-trapping effort per 100 trap-nights (*i*.*e*., 100 nights that the camera-trap was operational). To characterize the anthropogenic pressures across our study sites, we allocated images of humans recorded in camera-trap photos into one of the following categories: (1) “bird catchers”, as indicated by people carrying bird cages and other apparent bird-catching tools; (2) “encroachers”, which are typically individuals unaccompanied by specialized equipment; (3) “loggers”, who brought logging tools of various sort (*e*.*g*., saws and axes), or were photographed carrying logs; (4) “poachers”, who typically carried weapons used in hunting; (5) “fishermen,” who carried fish and/or visible fishing tools, such as fishing rods or nets; and (6) “non-timber forest product (NTFP) collectors,” usually depicted carrying NTFP products or harvesting tools, such as machetes and long bamboos, to collect forest fruits.

### Modelling occupancy and spatial overlap

We used single-season occupancy modeling analyses [31] to explore those factors influencing habitat use by carnivores and potential prey species. Among the important covariates we examined were altitude (*m* asl; as recorded in the field at each station), distance to the nearest forest edge (*m*), distance to the nearest road (*m*), and distance to the closest big river (*m*). We obtained forest cover layer based on the interpretation of satellite imagery data from Eyes on the Forest [32], and subsequent ground verification surveillance. Data on road and river networks were obtained from Badan Informasi Geospasial 2013 [33]. Details of all map layers were projected onto the Universal Transverse Mercator (UTM) under the World Mercator. All spatial representations and related analyses were conducted in ArcGIS 10.4 [26].

We hypothesized that shorter distances to roads, big rivers, and forest edges would lead to decreased independent occurrence records of carnivores and prospective prey species. We also assumed lower elevations would be related to higher human disturbance. A preliminary analysis using Pearson’s correlation demonstrated that the distance to roads, and the distance to forest edges, were strongly correlated with each other (*r* = 0.79); we therefore did not include these two covariates together in the same model (S2 Table). We also included survey effort (*e*.*g*., no. of camera-trap nights per station) as a covariate in our detection probability models [*p*(Effort)]. We used Akaike Information Criteria (AIC) as our multi-model inference and selection framework, top models for which were indicated by relatively low AIC values [34]. We used the corrected version of AIC (AIC_c_) for smaller sample sizes, and conducted model averaging for our top models, *i*.*e*., AIC_c_ ≤ 2 [35]. Those covariates associated with top models for single-species occupancy models informed the development of two-species occupancy models.

We used a single-season two-species occupancy model [18] to investigate the spatial occurrences and relationships between presumably dominant species (*i*.*e*., people and tigers), as well as presumably “subordinate” species (*i*.*e*., as represented by the other carnivores and their prey species). We hypothesized that carnivores and their prey would likely avoid the presence of humans, and larger competitors or predators. We did not use a multi-species occupancy model, as such models assume symmetrical interactions [11, 36], and we do not believe this to be realistic. We treated potential prey species as “dominant” species, as they likely influenced the occupancy of tigers and other carnivores. We also assessed Species Interaction Factors (SIF) estimated from the conditional two-species occupancy model, where SIF = ψ^A^ × ψ^BA^ / (ψ^A^ × (ψ^A^ × ψ^BA^ + (1 − ψ^A^) × ψ^Ba^)) [18], ψ^A^ = the probability of occupancy for species A, ψ^BA^ is the probability of occupancy of species B given species A is present, and ψ^Ba^ is the probability of occupancy for species B given species A is absent. Paired species occurrences were considered independent when SIF = 1, overlapped when SIF > 1, and were non-overlapping when SIF < 1. We generated SIFs and 95% CI using *package ‘mvtnorm’ version 1*.*1–1* in *R version 3*.*6*.*3* [37].

Detection history was based on distinct survey occasions, each of which consisted of a 14-day interval; positive detections for each survey occasion were indicated by a ‘1’, whereas a lack of detections, or failure to detect, was represented by a ‘0’ (*i*.*e*., non–detections). A ‘-’ indicated a camera-trap station that was not active during the specified time interval [19]. We performed all occupancy modelling in the *package ‘wiqid’ version 0*.*3*.*0* in *R version 3*.*6*.*3* [38].

### Temporal patterns and overlap

Carnivores and their prey species tend to adapt their daily temporal activity, presumably to avoid competitors and predators, respectively [19]. We used *package ‘overlap’ version 0*.*3*.*2* in *R version 3*.*6*.*3* to calculate Kernel Density Estimators (KDE) and quantify the degree of overlap in activity patterns between people and large carnivore species. Specifically we focused on three Δ estimators, which ranged from 0 (no overlap) to 1 (complete overlap) [17, 39, 40]. The ${\hat{\Delta }}_{4}$ represents the best estimator when the smallest sample included at least 50 observations; for smaller sample sizes, we used ${\hat{\Delta }}_{1}$ [39]. After performing smoothed bootstraps of 10,000 iterations [39], we classified activity overlap of Δ ≤50th percentile as “low”, activity overlap in the 50th < Δ <75th percentile as “moderate”, and activity overlap of the Δ > 75th percentile as “high” [40]. To test for differences in activity patterns among study sites, we used the nonparametric circular Mardia–Watson–Wheeler statistical test [41, 42].

## Results

We recorded a total of 14,013 camera-trap nights across 147 active camera-trap stations and an effective sampling area of 935 km^2^. Our largest survey efforts were in Southern Rimbang Baling, where we accumulated 3,269 trap nights from 32 camera-trap stations across 208 km^2^. This was the most isolated of our study sites and had the longest mean distances to forest edges (7,731 *m*; range 3,156–12,399 *m*), roads (10,667 *m*; range 3,655–16,234 *m*), and to large rivers (7,731 *m*; range 3,156–12,399 *m*). Conversely, Tesso Nilo was the most accessible site and had the lowest mean elevation (81 *m* asl; range 41–110 *m* asl), shortest mean distance to roads (307 *m* asl; range 2–1,675 *m*), and the shortest mean distance to forest edges (1,577 *m*; range 0–4,380 *m*) (Table 1). Across all sites, we obtained a total of 1,818 (37.59%) independent photographs of people appearing to be engaged in illegal activities, representing 37.6% of all camera-trap images from our study. Malayan sun bears were by far the most frequently detected medium-sized or large carnivore across all camera-trap stations (n = 565), followed by Sunda clouded leopards (n = 190). The most frequently detected potential prey species across all camera-trap stations was the southern red muntjac (n = 658); in contrast, the fewest number of detections were for sambar (n = 15) (S4 Table). Among all study sites, Tesso Nilo experienced the most intense anthropogenic pressures as indicated by our records of logging-related activities (515 independent events or 61%; CTR = 21.56/ 100 trap nights; S5 Table), and people with motorbikes (689 independent photographs or 79%; CTR = 28.79/ 100 trap nights; S5 Table). Based on our data across all sites, Rimbang Baling and Bukit Bungkuk were subject to relatively lower anthropogenic pressures (*i*.*e*., based on our total CTR of people).

**Table 1:** Survey efforts and characteristics of all study sites within four major protected areas.

Survey Characteristics	Northeastern Rimbang Baling	Northwestern Rimbang Baling	Southern Rimbang Baling	Bukit Bungkuk	Bukit Betabuh	Tesso Nilo	Entire study area
**Survey effort**							
**Area status**	Wildlife reserve	Wildlife reserve	Wildlife reserve	Nature reserve	Protected forest	National park	
**Survey period (dd/mm/yyyy)**	16/11/2011–25/02/2012	12/02/2014–10/06/2014	28/08/2015–19/12/2015	09/06/2012–17/09/2012	11/01/2013–25/04/2013	18/07/2013–02/11/2013	
**Effective trap nights**	1,688	3,169	3,268	1,762	1,791	2,335	14,013
**Trap polygon size (km** ^**2**^ **)** ^a^	95	195	208	99	161	177	935
**Camera stations**	20	31^b^	32	20	20	25	148
**Camera loss**	0	4	0	0	0	1^c^	5
**Habitat variables (mean (min–max))**							
**Mean altitude with min–max (*m* asl)** ^d^	289 (102–830)	746 (378–1,247)	559 (291–886)	328 (158–572)	333 (64–580)	81 (41–110)	421 (35–1,274)
**Mean distance to roads with min–max (*m*)** ^e^	4,489 (1,303–9,026)	6,324 (2,083–1,0487)	10,667 (3,655–16,234)	4,988 (921–7,768)	3,504 (200–7,649)	307 (2–1,675)	5,429 (2–16,234)
**Mean distance to big rivers with min–max (*m*)** ^e^	9,762 (4,709–13,895)	2,162 (199–5,422)	10,702 (4,229–14,960)	6,239 (1,119–10,600)	4,320 (821–8,053)	4,257 (746–9,386)	6,258 (199–14,960)
**Mean distance to edge of forests with min–max (*m*)** ^f^	2,359 (238–4,986)	4,993 (752–9,208)	7,731 (3,156–12,399)	2,591 (285–6,076)	1,627 (0–4,138)	1,577 (0–4,380)	3,881 (0–12,399)

^a^based on Minimum Convex Polygon (MCP) of camera stations.

^b^there were only 30 active stations due to camera theft.

^c^burned due to forest fires.

^d^manually derived from GPS waypoints at camera-trap stations.

^e^using Badan Informasi Geospasial (BIG) data [33].

^f^using land cover map 2012 [32].

### Occupancy and habitat use models

**Fig 1:**
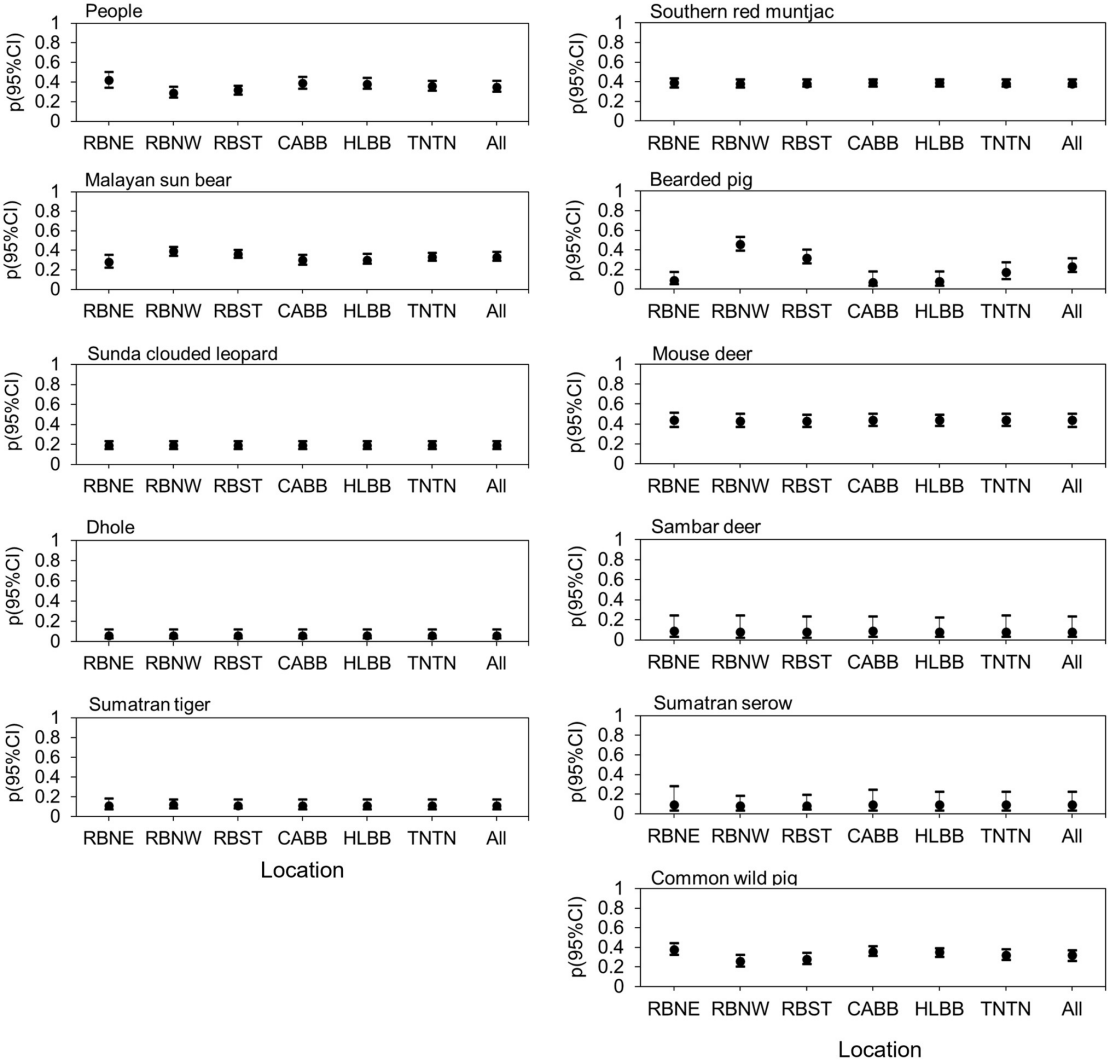
Detection probability with 95% CI based on model averages for top models with ΔAICc ≤ 2: People, large carnivores, and potential prey species for 147 camera stations across all study sites. RBNE, Northeastern Bukit Rimbang Bukit Baling; RBNW, Northwestern Bukit Rimbang Bukit Baling; RBST, Southern Bukit Rimbang Bukit Baling; CABB, Bukit Bungkuk; HLBB, Bukit Betabuh; TNTN, Tesso Nilo; All, “All study sites”.

We documented large variations in our naive occupancy estimates for large carnivores, potential prey species, and people across all study sites. Among all sites, the highest naive occupancy estimates for sun bears occurred in Southern RB (0.91 ± SD 0.30), for Sunda clouded leopards in Northwestern RB (0.77 ± SD 0.43), and for tigers in Southern RB (0.50 ± SD 0.51), relative to other study areas (S6 Table). However, our naive occupancy estimate for dholes (0.09 ± SD 0.30) was low across all study areas. Among all potential prey for all study sites, the highest naive occupancy was for the southern red muntjac (0.84 ± SD 0.37), whereas the lowest was for the Sumatran serow (0.07 ± SD 0.25), a species that we only recorded in Rimbang Baling. Across all study sites, mean naive occupancy for people was relatively high (0.60 ± SD 0.49) (S6 Table). Mean detection probability for people across all study sites (0.35; 95% CI: 0.30–0.41) was also higher than for each large carnivore, with the highest mean detection probability for humans originating from Northeastern Rimbang Baling (Fig 1). Whereas sun bears had the highest detection probability among all large carnivores (0.33; 95% CI: 0.29–0.38), dholes had the lowest (0.06; 95% CI: 0.03–0.12) (Fig 1).

**Fig 2:**
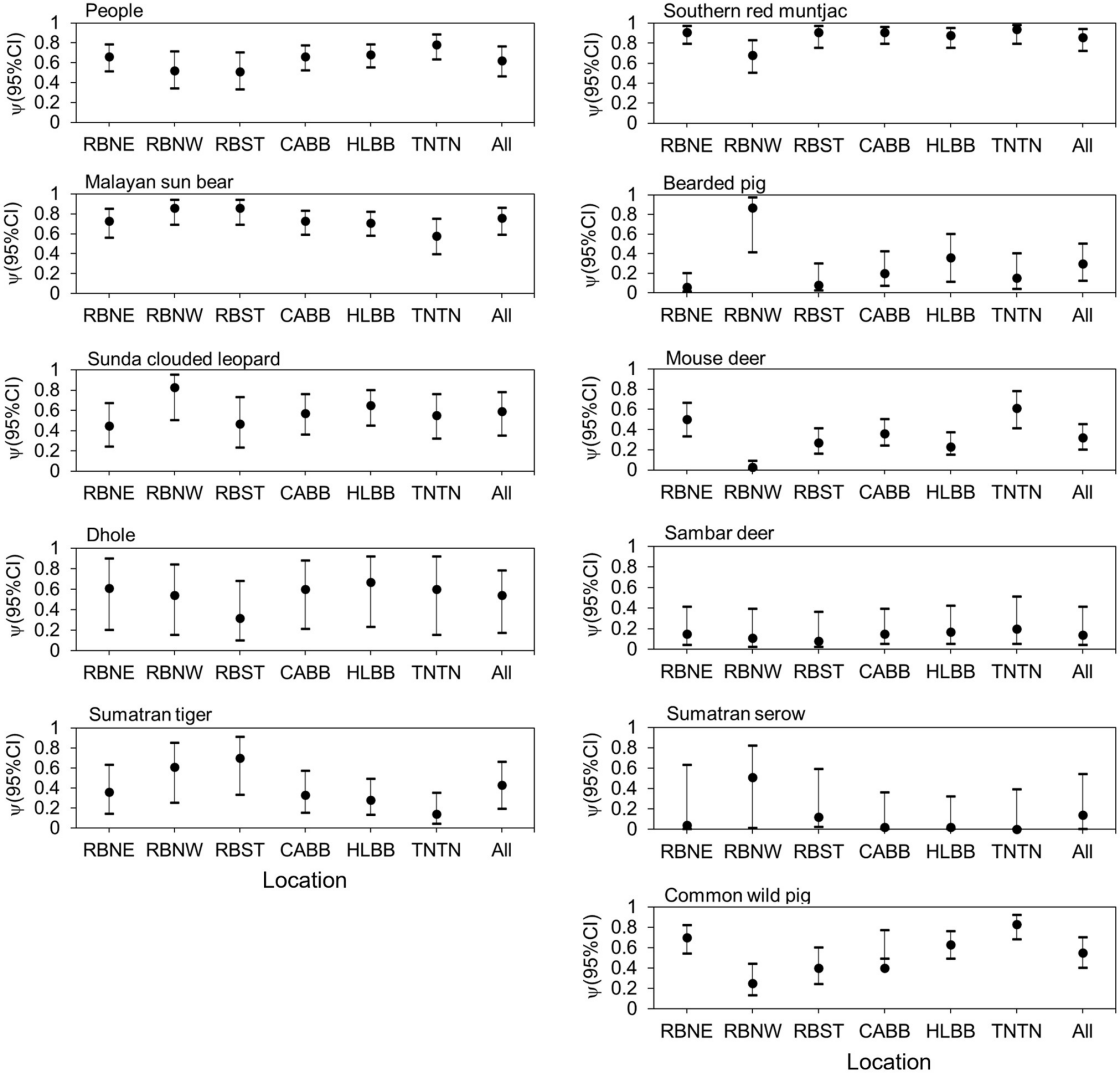
Probability of occupancy (ψ) with 95% CI based on model averages for top models with ΔAICc ≤ 2: People, large carnivores, and potential prey species for 147 camera stations across all study sites. RBNE, Northeastern Bukit Rimbang Bukit Baling; RBNW, Northwestern Bukit Rimbang Bukit Baling; RBST, Southern Bukit Rimbang Bukit Baling; CABB, Bukit Bungkuk; HLBB, Bukit Betabuh; TNTN, Tesso Nilo; All, “All study sites”.

The average modeled occupancy estimates for people across all sites combined was ψ = 0.62 (95% CI: 0.46–0.76), with estimates for Tesso Nilo higher relative to all other sites (0.78, 95% CI: 0.63–0.88) (Fig 2). Perhaps unsurprisingly, Tesso Nilo also had the lowest ψ for sun bears, clouded leopards, and tigers. Among all carnivores and across all study sites overall, sun bears had the highest mean ψ (0.76; 95% CI: 0.39–0.75), whereas tigers had the lowest (ψ = 0.43, 95% CI 0.04–0.35) (Fig 2). Among all potential prey species and across all study sites, southern red muntjac had the highest mean occupancy (ψ = 0.86; 95% CI: 0.72–0.94), followed by wild boar (ψ = 0.55; 95% CI: 0.40–0.70). Sambar deer, the ungulate with the largest body size, had a very low probability of occupancy overall (ψ = 0.14; 95% CI: 0.04–0.54) (Fig 2c).

We used β estimates from the model with the lowest AIC_c_ value to identify the most important factors influencing habitat use of large carnivores, potential prey species and humans; significance was indicated by β estimates not overlapping with zero. For example, elevation had a strong negative or inverse relationship to human presence (β_Alt_ = -0.51; 95% CI: -0.44 –-0.66), but was positively correlated for Malayan sun bear ψ (β_Alt_ = 0.69; 95% CI: 0.20–1.18) and more strongly for Sumatran serows (β_Alt_ 2.46; 95% CI: 0.56–4.35) (S8 Table). The occurrence of southern red muntjac and mouse deer was strongly associated with distances farther from roads (β_DistRoad_ = 0.92; 95% CI 0.08–1.77, and β_DistRoad_ = 1.23; 95% CI: 0.40–2.07, respectively).

**Fig 3:**
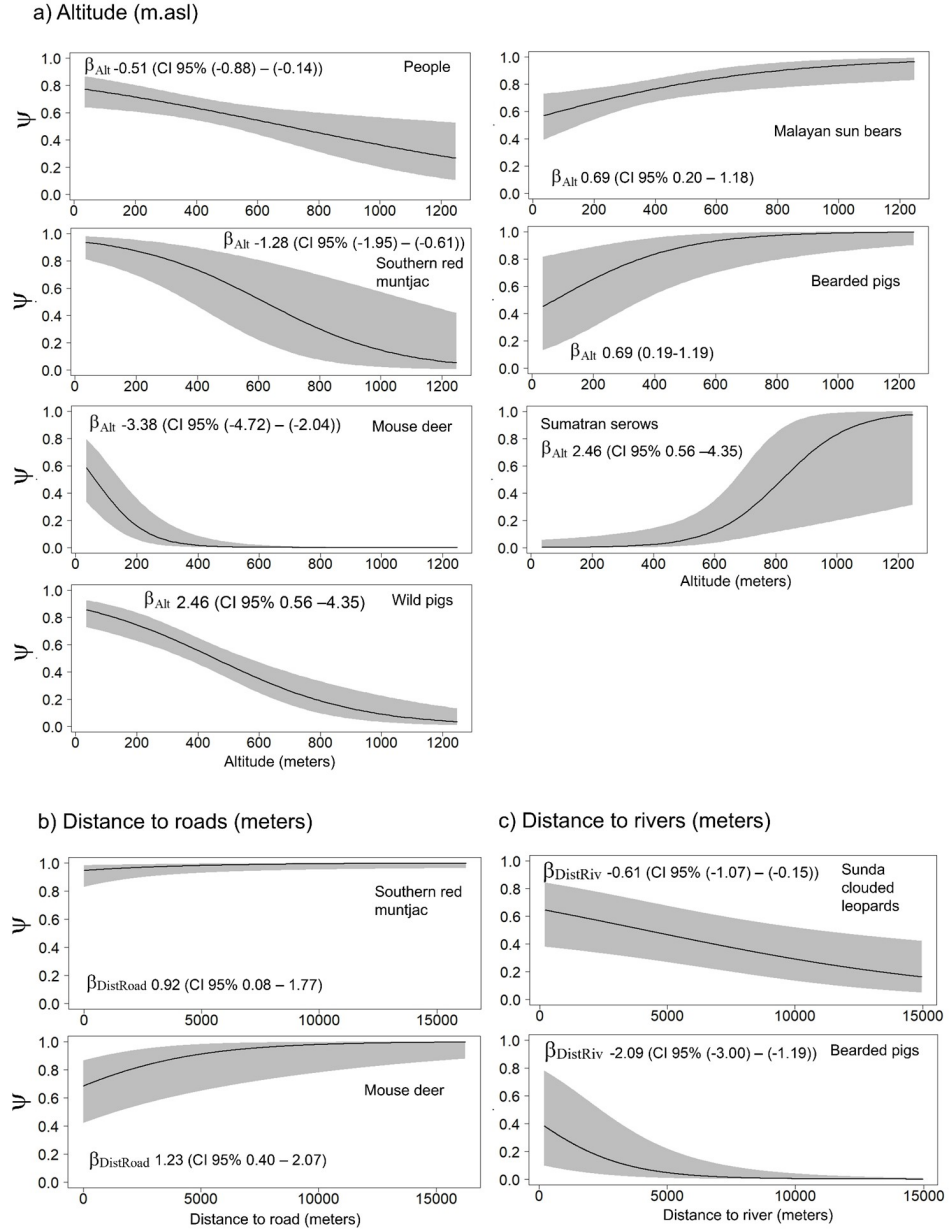
Relationships of habitat covariates to the probability of occupancy for all study sites as selected from models with the smallest AIC_c_. Correlations are based on a robust *β* as indicated by the ±95% CI not overlapping zero.

Across all study sites, human occupancy (ψ) decreased from approximately 0.8 in lower elevation areas, to 0.3 for the highest elevation areas. Malayan sun bear ψ was higher in lower altitude areas (Fig 3), whereas ψ of clouded leopards was higher at farther distances from big rivers (Fig 3). For prospective prey species like southern red muntjac, mouse deer, and bearded pigs, altitude had a strong negative influence on ψ. In contrast, Sumatran serows tended to have higher occupancy rates at higher altitudes. Both southern red muntjac and mouse deer had higher occupancy estimates farther from roads (Fig 3).

### Spatial overlap

We generated naive spatial overlap estimates of two-species pairs by examining occasions when and where they occurred together at given camera-trap stations. In a few cases, we detected high naive spatial overlap (> 0.50) between people and large carnivores, including: 1) between people and Malayan sun bears at three study sites (Rimbang Baling Northeastern, Rimbang Baling Northwestern, and Tesso Nilo); 2) and, between people and Sunda clouded leopards in Rimbang Baling Northeastern. We also recorded high naive spatial overlap (> 0.50) between people and southern red muntjac in RB Northeastern and RB Northwestern, and between people and bearded pigs in RB Northeastern (S6 Table).

We also investigated the ψ of large carnivores and potential prey species in the context of human and tiger presence and absence. The ψ of both dholes and tigers was lower when people were absent, whereas the occupancy of Sunda clouded leopards was higher in the absence of people (S10 Table). When tigers were present on the landscape, the ψ of the three other large carnivores overall tended to be higher (S11 Table). For prey species, two-species occupancy models only performed well for southern red muntjac and wild boar; this was largely because there were sufficient samples and occasions for both species to estimate spatial overlap with both people and tigers. The ψ of these two prospective prey species was similar in all study sites when people were present (S12 Table).

**Fig 4:**
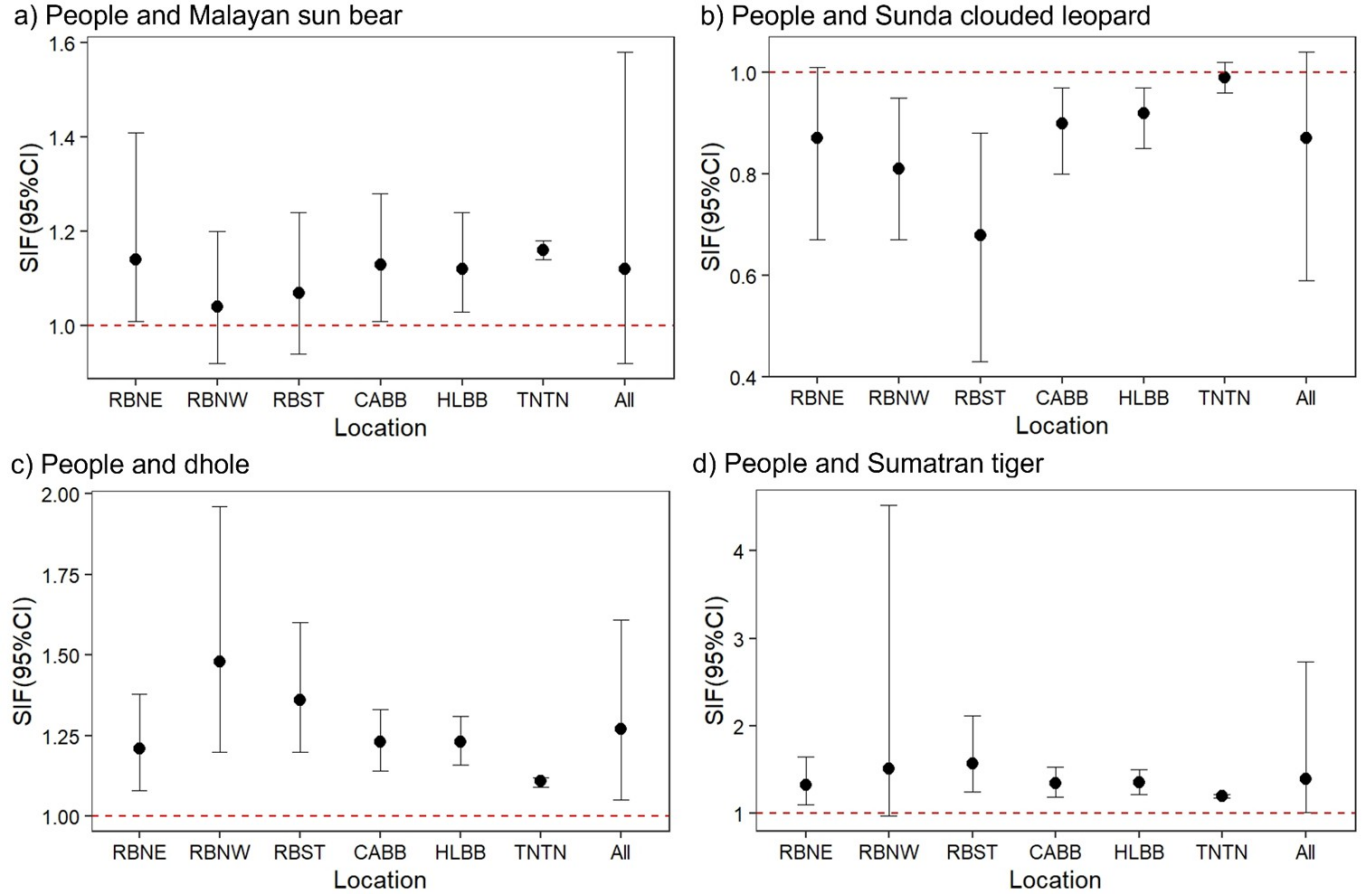
Spatial overlap between people (dominant, species A) and large carnivores (subordinate, species B) based on model-averaged ΔAIC_c_ ≤ 2 for 147 camera-trap stations across all study sites. A strong SIF is indicated by the 95% CI not overlapping with “1”; RBNE, Northeastern Bukit Rimbang Bukit Baling; RBNW, Northwestern Bukit Rimbang Bukit Baling; RBST, Southern Bukit Rimbang Bukit Baling; CABB, Bukit Bungkuk; HLBB, Bukit Betabuh; TNTN, Tesso Nilo; All, “All study sites”.

**Fig 5:**
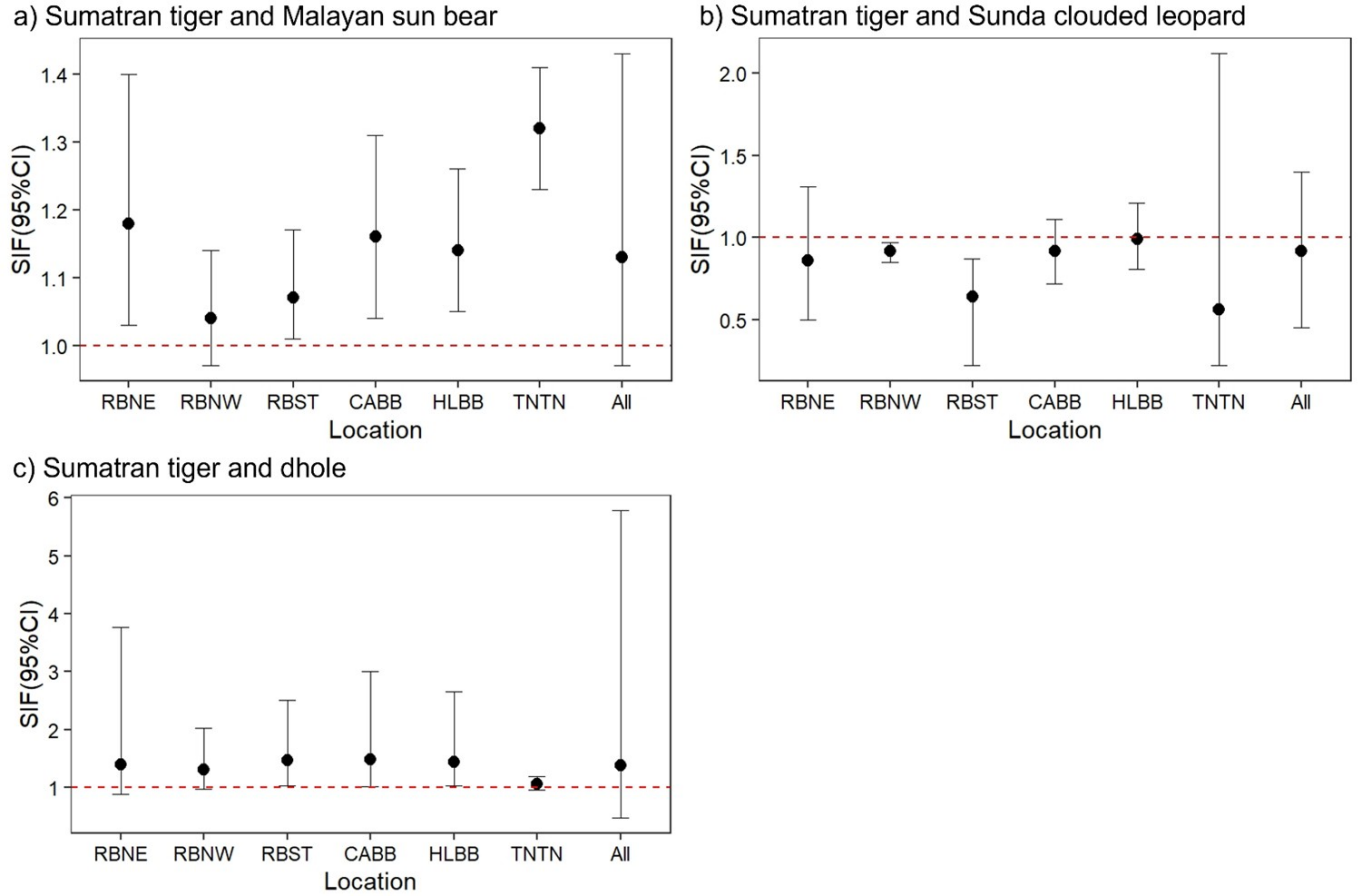
Spatial overlap between Sumatran tigers (dominant, species A) and other large carnivores (subordinate, species B) based on model-averaged ΔAICc ≤ 2 for 147 camera-trap stations across all study sites. A strong SIF is indicated by the 95% CI not overlapping with “1”; RBNE, Northeastern Bukit Rimbang Bukit Baling; RBNW, Northwestern Bukit Rimbang Bukit Baling; RBST, Southern Bukit Rimbang Bukit Baling; CABB, Bukit Bungkuk; HLBB, Bukit Betabuh; TNTN, Tesso Nilo; All, “All study sites”.

**Fig 6:**
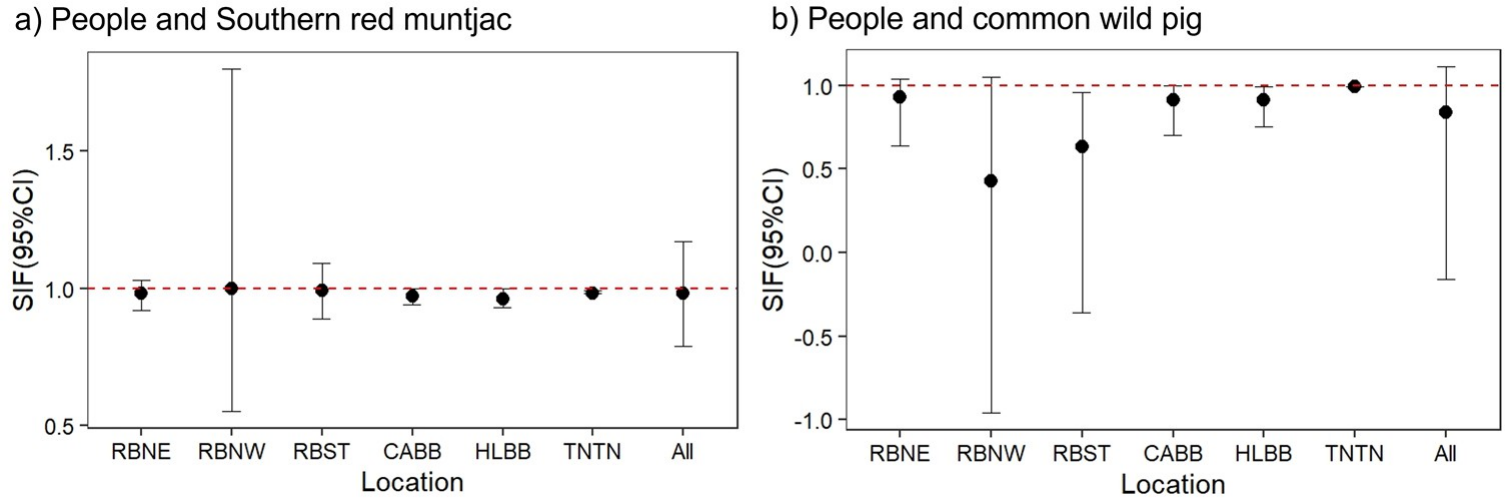
Spatial overlap between people (dominant, species A) and potential prey (subordinate, species B) based on model-averaged ΔAIC_c_ ≤ 2 for 147 camera-trap stations across all study sites. A strong SIF is indicated by the 95% CI not overlapping with “1”; RBNE, Northeastern Bukit Rimbang Bukit Baling; RBNW, Northwestern Bukit Rimbang Bukit Baling; RBST, Southern Bukit Rimbang Bukit Baling; CABB, Bukit Bungkuk; HLBB, Bukit Betabuh; TNTN, Tesso Nilo; All, “All study sites”.

**Fig 7:**
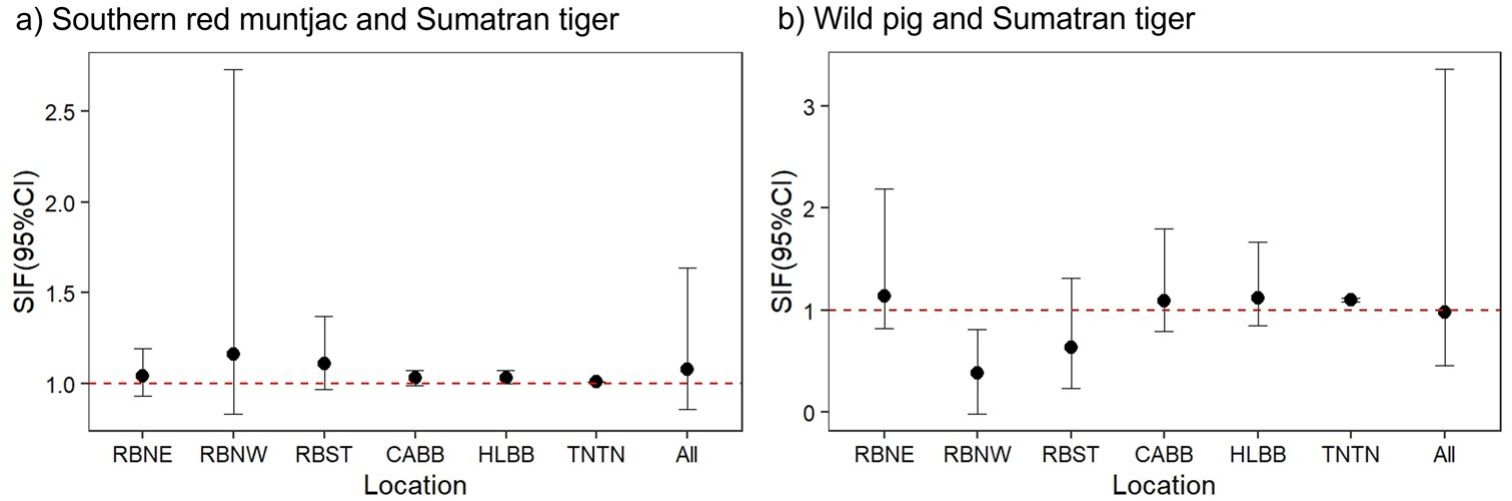
Spatial overlap between potential prey species (dominant, species A) and Sumatran tigers (subordinate, species B) based on model-averaged ΔAICc ≤ 2 for 147 camera-trap stations across all study sites. A strong SIF is indicated by 95% CI not overlapping with “1”; RBNE, Northeastern Bukit Rimbang Bukit Baling; RBNW, Northwestern Bukit Rimbang Bukit Baling; RBST, Southern Bukit Rimbang Bukit Baling; CABB, Bukit Bungkuk; HLBB, Bukit Betabuh; TNTN, Tesso Nilo; All, “All study sites”.

We considered spatial overlap to be “strong” or significant (SIF > 1), non-existent or “avoidant” (SIF < 1) if 95% CIs did not include “1”. Our top models indicated support for strong spatial co-occurrence between people and sun bears, between people and dholes, and between people and tigers (Fig 4). This was true even though some CIs included “1” (*i*.*e*., a weak or non-significant SIF). Sun bears and dholes also exhibited significant spatial overlap with tigers (SIF > 1) (Fig 5), whereas Sunda clouded leopards significantly avoided (SIF < 1) both people and tigers (Figs 4 and 5). Southern red muntjac and bearded pigs exhibited only weak spatial overlap with people and tigers, *i*.*e*., a 95% CI for the SIF including “1” (Figs 6 and 7). Southern red muntjac also appeared to be neutral to people (SIF was approximately “1”), whereas wild boar exhibited weak avoidance of people (SIF < 1) (Fig 6).

### Temporal patterns and overlap

**Fig 8:**
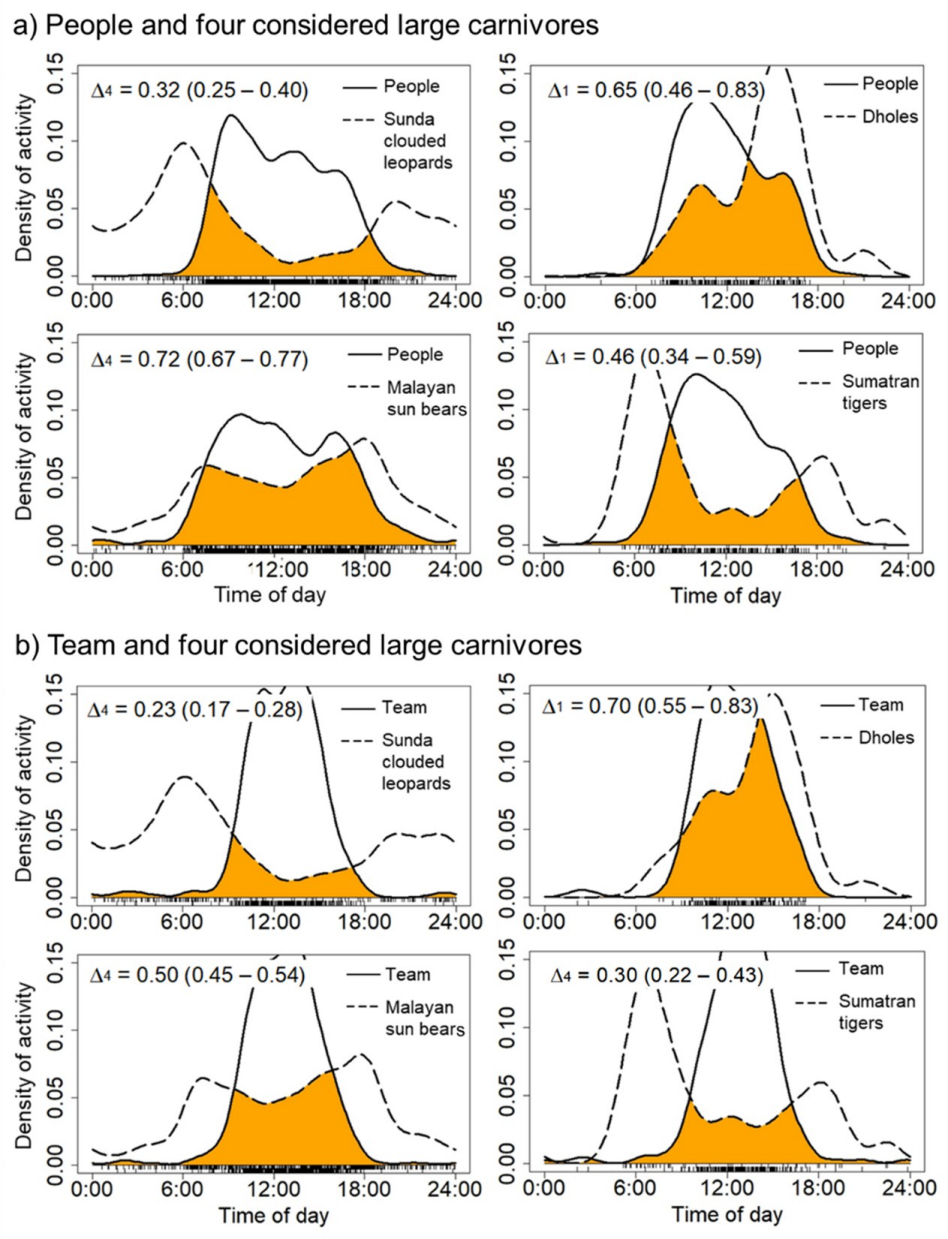
Estimates of the daily activity patterns of people, team and four large carnivores in all study sites. The overlap coefficient is indicated by the shaded orange area in each plot with the estimate of overlap (**Δ)** and 95% CI.

**Fig 9:**
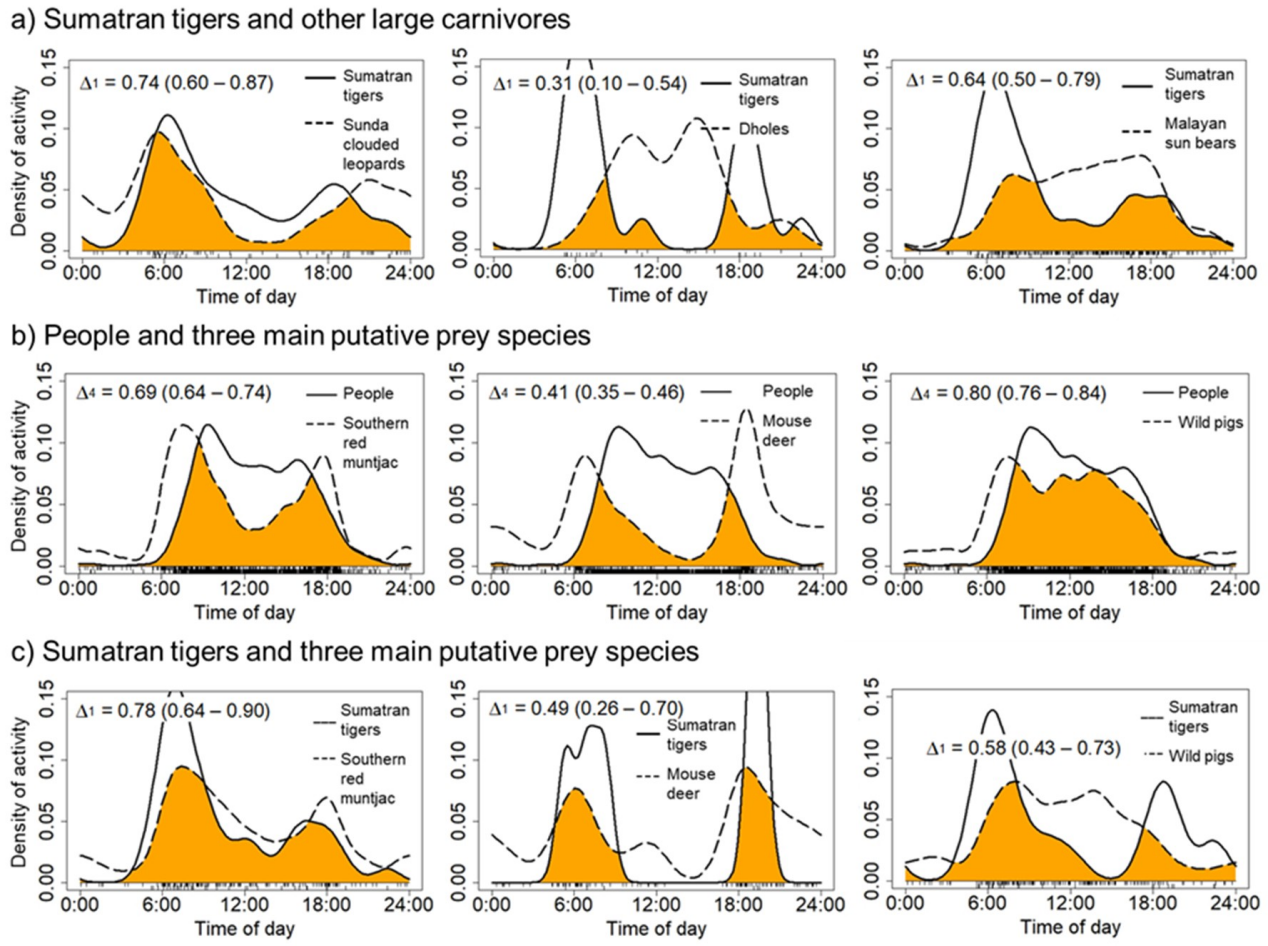
Estimates of the daily activity patterns of people, Sumatran tigers, three other large carnivores, and three main potential prey species. The overlap coefficient is indicated by the shaded orange area in each plot with the estimate of overlap (**Δ)** and 95% CI.

The Kernel Density Estimation (KDE) revealed that sun bears and dholes exhibited moderate temporal overlap with people across all study sites (Δ = 0.74; 95% CI: 0.67–0.77, and Δ = 0.65; 95% CI: 0.46–0.83, respectively) (Fig 8); however, both species exhibited strong temporal overlap with people in Northeastern Rimbang Baling (Δ = 0.79 and 0.80, respectively; Table 2). In contrast, tigers and clouded leopards exhibited low temporal overlap with people (Fig 8), but relatively high temporal overlap with each other (Δ = 0.74; 95% CI: 0.60–0.87) (Fig 9). Southern red muntjac activity appeared to strongly overlap with people across all study sites (Fig 9). In addition, people and wild boar exhibited high temporal overlap with each other (Δ = 0.80, 95% CI: 0.76–0.84), and southern red muntjac exhibited high temporal overlap with tigers (Δ = 0.78, 95% CI: 0.64–0.90) across all study sites (Fig 9). Overall, the slight to large majority of records of people, dholes, sun bears, and tigers occurred during the day (*i*.*e*., between 07h00 to 17h00: 85.86%, 86.49%, 55.75% and 51.47% of observations, respectively; S14 Table). We also found that the activity patterns of people, sun bears, and mouse deer, were significantly different for all sites (Mardia–Watson–Wheeler test: *p < 0*.*05*) (S14 Table).

**Table 2:** Estimates of temporal overlap (Δ) with 95% bootstrap confidence interval of people, team, large carnivores, and potential prey species for all study sites.

Pairing species		Δ (95% CI)					
		Northeastern Rimbang Baling	Northwestern Rimbang Baling	Southern Rimbang Baling	Bukit Bungkuk	Bukit Betabuh	Tesso Nilo
People and	Sunda clouded leopards	0.36 (0.23–0.50)	0.32 (0.20–0.45)	–	–	0.16 (0.00–0.36)	0.49 (0.36–0.63)
	Dholes	0.79 (0.48–1.00)	0.51 (0.20–0.80)	0.27 (0.00–0.63)	–	–	0.27 (0.20–0.44)
	Malayan sun bears	0.80 (0.68–0.90)	0.69 (0.59–0.79)	0.53 (0.38–0.69)	–	–	0.54 (0.43–0.66)
	Sumatran tigers	0.33 (0.11–0.57)	0.50 (0.25–0.74)	0.58 (0.33–0.81)	–	0.22 (0.05–0.39)	–
Team and	Sunda clouded leopards	0.19 (0.09–0.29)	0.21 (0.13–0.31)	0.21 (0.03–0.44)	0.32 (0.11–0.55)	0.16 (0.01–0.35)	0.16 (0.04–0.29)
	Dholes	0.54 (0.24–0.81)	0.66 (0.38–0.88)	0.08 (-0.02–0.26)	0.81 (0.55–0.99)	–	0.81 (0.70–0.99)
	Malayan sun bears	0.51 (0.41–0.64)	0.53 (0.46–0.61)	0.40 (0.31–0.50)	0.41 (0.27–0.55)	–	0.35 (0.26–0.47)
	Sumatran tigers	0.18 (0.00–0.38)	0.42 (0.22–0.61)	0.42 (0.19–0.65)	–	0.10 (-0.03–0.26)	0.10 (0.09–0.18)
Tigers and	Sunda clouded leopards	0.69 (0.46–0.88)	0.50 (0.32–0.68)	–	–	–	–
	Dholes	0.28 (0.00–0.60)	–	–	–	–	–
	Malayan sun bears	0.38 (0.15–0.62)	0.70 (0.53–0.86)	0.37 (0.20–0.54)	–	–	0.37 (-0.10–0.74)
People and	Southern red muntjac	0.64 (0.52–0.76)	0.63 (0.50–0.75)	0.54 (0.40–0.68)	0.54 (0.24–0.80)	0.75 (0.64–0.85)	0.73 (0.65–0.81)
	Mouse deer	0.49 (0.63–0.86)	–	0.24 (0.08–0.41)	–	0.28 (0.19–0.37)	0.54 (0.43–0.65)
	Wild pigs	0.79 (0.71–0.87)	0.83 (0.57–1.02)	0.66 (0.41–0.87)	–	0.81 (0.71–0.88)	0.77 (0.67–0.85)
Tigers and	Southern red muntjac	0.67 (0.39–0.90)	0.62 (0.38–0.83)	0.60 (0.42–0.75)	–	0.38 (0.13–0.65)	0.44 (0.19–0.70)
	Mouse deer	0.26 (-0.01–0.53)	–	–	–	–	0.11 (-0.15–0.38)
	Wild pigs	0.50 (0.27–0.70)	0.41 (0.06–0.82)	–	–	0.36 (0.14–0.61)	0.07 (-0.15–0.32)

## Discussion

Our study investigated the relationship between people, large carnivores, and potential prey species by assessing their spatiotemporal overlap and habitat use across human-disturbed habitats in key protected areas of central Sumatra. Our results serve as important reminders as to the influence of humans on the ecology of Sumatra’s terrestrial medium-large mammals, an issue of increasingly pressing concern across our study sites given the increased encroachment of people we recorded. Our use of camera traps, which are typically more efficient than traditional sampling methods (*e*.*g*., direct observation, radio telemetry) in collecting continuous data simultaneously for many mammal species [43, 44], resulted in novel findings about the impact of human presence on Sumatran carnivores and their prey. Our study also complements and corroborates the findings of prior regional studies on these species [45, 46].

Because funding, logistics, and manpower for our study were limited, our sampling effort could not fully account for the seasonal or annual dynamics of animal movements and distributions, and the impact of human disturbance. We therefore suggest that our study results must be interpreted with caution, particularly with respect to conclusions outside the parameters of our study. For example, Tesso Nilo NP, which was the most disturbed area among all sampling locations in 2013, incurred greater human disturbance in ensuing years. This likely influenced large carnivore and prey occupancy further, but unfortunately, we were not able to evaluate this.

### Occupancy and habitat use models

We found that human occupancy, as indicated by both naive and modelled occupancy estimates, and human detection probability, was very high across our study areas. This suggests the recurring occurrence of large-scale, unlawful human intrusions into protected areas, especially at lower elevation sites like Tesso Nilo National Park. We also note that human occurrence in our study was also relatively higher than was recorded for other similar studies in Sumatra, such as in Bukit Barisan Selatan National Park (naive occupancy = 0.33) [24] and Kerinci National Park, where the highest occupancy probability of any sampled site was ψ = 0.43 [25]. However, we also believe people tended to deliberately avoid camera traps, which may have caused a negative bias in the probability of detection. We therefore suggest our parameter estimates for people are conservative. In addition, we photographed people covering their faces in front of cameras across our study sites. We also endured vandalism and theft of camera-traps, including the loss of units from Northwestern Rimbang Baling (four units stolen) and Tesso Nilo (*e*.*g*., one unit burned during man-made forest fires). Human activity also varied overall among study sites. Lower elevation areas, such as Tesso Nilo NP, were subject to the greatest levels of anthropogenic disturbance. Tesso Nilo is known to be “highly threatened” by people, as > 70% of natural forests in that area have been illegally converted to, or impacted by, agricultural plantations [47].

We also confirmed the presence of the four large and medium-sized carnivores at each of the study sites. Malayan sun bears had the highest estimated probability of occupancy among all large carnivores and across all study sites. Interestingly, a prior study from Kerinci NP concluded that the highest sun bear occupancy actually occurred in degraded forest [48]. Unlike tigers, dholes, and clouded leopards, all of which are obligate carnivores and frequently evaluated with respect to potential prey species [49–51], sun bears are opportunistic and omnivorous “carnivores”. They can access diverse foods, including termites, ants, beetle larvae, stingless bee larvae, honey, and many species of fruit [52]. However, the distribution and habitat use of sun bears is still likely determined by food availability, including the density of fruiting trees [53]. Although tigers faced greater local threats from poaching and retaliatory killing, previous studies have suggested there was no or little trapping and poaching of sun bears on Sumatra [7, 53].

Our study also underscores the dynamic nature of large carnivore habitats in Sumatra [46], particularly the differential importance of large-scale habitat features to carnivores and their prey. Elevation, and proximity to forests, roads, and big rivers, all had varying impacts on our target species; these effects all have management implications for the maintenance of ecosystem diversity [6]. Sunda clouded leopards for example preferred areas farther from big rivers, possibly because such rivers might have experienced greater human disturbance. These findings are also consistent with clouded leopards “avoiding” human presence in our study areas (SIF < 1). Other studies have concluded that water bodies were not barriers to the movements of Sunda clouded leopards occupying peat-swamp forests, and that similarly, clouded leopard occupancy was greater at farther distances from rivers [54, 55]. Although other studies in Sumatra have recorded Sunda clouded leopards using human-impacted habitats, overall they appear to avoid humans disturbances, occurring more at farther distances from forest edges, and at slightly higher elevations [25, 56]. Big rivers, areas closer to forest edges, and lowlands, are subject to greater human disturbances, and may facilitate access and travel routes for the people at our study sites. A study in Kerinci Seblat corroborated that Sunda clouded leopards preferred areas with dense tree cover, where human activities were also lower than among non-forested areas [55].

Despite that our habitat use models did not suggest the presence of strong covariates associated with tiger occurrence, other studies concluded that tiger occurrence was inversely correlated with distance-to-public roads (*i*.*e*., tigers occurred closer to roads). However, tiger occurrence was also dependent on large contiguous forest blocks in Kerinci and Riau Province [46, 57]. Likewise, another prior study found that, based on lower CTR, tigers strongly used areas farther from big water bodies in the peatlands of Kerumutan, with zero tiger records from the peatlands of Kampar Peninsula [22].

Dholes were detected in more forested areas than otherwise, which was consistent with some preliminary findings for the same region [58]. However, we found that proximity to the forest edge received only weak inverse support (i.e., negative β) because the 95% CI overlapped with zero; this suggested that dhole occurrence was not significantly associated with forest cores more than forest edges. Similarly, forest cover also did not appear to impact the occurrence of other large carnivores, although this may reflect in part the degradation these habitats have endured in recent years. That dholes were detected so rarely overall in our study should be of great concern to their viability in this region.

Sambar deer have been recognized as a principal prey species for tigers, especially across Southeast Asia [2, 59, 60]. Our occupancy estimates for sambar were very low overall, with a naive estimate of 0.07 (± SD 0.25), and a ψ = 0.14 (95% CI 0.04–0.41). We note that this was much lower than for all other potential prey species. Sambar deer are threatened directly by poaching, as it is one of the most prized “wild meat” or bushmeat in Sumatra, mostly because of its large size [29, 61]. The low occupancy estimate of sambar deer in our study therefore is worrisome, and suggests both tiger and dhole populations might be severely constrained by low principal prey abundance well into the future unless populations are somehow buoyed.

Southern red muntjac and bearded pigs probably served as alternative main prey for tigers given the near absence of sambar, and both species were recorded widely across all study sites. At some sites, the measured spatial overlap between southern red muntjac and tigers supported a potential for interaction (*i*.*e*., SIF > 1). In Bukit Barisan Selatan NP, another study verified that southern red muntjac had a similarly widespread distribution, occupying 98% of the national park [24]. Southern red muntjac thus appear more resilient to hunting. Their relative abundance may still be relatively high in logged and other degraded forests, including in areas where the almost complete conversion of forests has occurred [62]. This is also despite that locals across central Sumatra, who are primarily Muslim, generally do not eat wild pigs as bushmeat [29], which likely places greater pressure on muntjac. Conversely, another study reported on substantial local demand for pigs by non-Muslim indigenous tribes, from which such meat is often exported to the other regions [28]. In addition, wild pigs in some areas may face heavier hunting pressures than southern red muntjac due to their propensity for crop-raiding and inflicting other conflict-related damage [28]. In contrast to wild boar, which were widespread across our study area, bearded pigs appeared to be distributed rather sparsely.

Our habitat models also suggested that the variation in occupancy and habitat use of prey species across our study sites were partly due to differences in elevation. For example, we only recorded the very rare Sumatran serow in Rimbang Baling, an area that is more varied topographically and thus may encompass more suitable habitat for this wild goat species. Serows are known to be more dependent on higher elevation forests and rough, hilly terrains that are difficult to access; not surprisingly, they are therefore less studied [63]. Bearded pig occupancy was also higher at higher elevation, which suggests that future regionwide and/or island wide studies may find more in these habitats. Conversely, the occupancy of muntjac, mouse deer and wild boar were all negatively correlated with elevation.

### Spatiotemporal overlap

We employed single season two-species occupancy models in order to generate conditional two-species occupancy models incorporating habitat covariates and SIF’s. A prior study investigating the spatiotemporal overlap between Sumatran tigers and prey species ranked potential prey species based on composite index scores of encounter or detection rates (i.e., CTR) [21]. This a less robust approach and also did not consider habitat covariates [21]. Our more approach using models integrating SIF’s showed strong evidence of spatial overlap between the following species pairings: people and sun bears, people and tigers, tigers and sun bears, tigers, and dholes (*i*.*e*., SIF > 1). We note that frequent human intrusion in large carnivore habitat could also habituate certain species to human presence, possibly explaining the relationship between people, sun bears, and tigers we found in our study. We point out this could be of future concern if threats like commercial poaching for the wildlife trade, and retaliatory killings resulting from conflict, were to suddenly emerge or increase.

Consistent with our occupancy-based findings, we found that Sunda clouded leopards appeared avoid humans (SIF < 1) as well as tigers. Further investigation into how Sunda clouded leopards successfully avoid tigers and people, including their ability to move in trees as a way to reduce spatial overlap, is recommended; these findings could have implications for their probability of detection in these contexts. They might also help explain further how or why Sunda clouded leopards are able to persist in areas of relatively high human disturbance [25, 49, 64, 65]. Like Sunda clouded leopards, Malayan sun bears are dependent on forests with high tree canopies; they did not appear to avoid tigers in our study, despite that tigers elsewhere are reported to prey on sun bears [2, 66, 67]. However, it is possible that the apparent overlap between tigers and sun bears, or any species pairing for that matter, is facilitated by elevational segregation, differential use of vertical strata, variation or heterogeneity in microhabitats, different activity patterns, and use of different prey or prey of different size [2]. Finally, whilst southern red muntjac tended to have neutral spatial overlap with people, SIF values for wild boar suggested slight avoidance of people. As discussed for our occupancy findings, this may be due to retaliation resulting from conflict in agricultural plantations, and/or hunting by non-Muslim locals and their dogs.

We must caveat that some limitations imposed on our conclusions about spatial overlap are due to our 14-day occasional survey period, which is a relatively coarse temporal interval. We proposed the use of a 14-day occasion periods to enhance binary sample sizes or occasions overall and offset the low detection rates for large carnivores across our study areas. This is not without precedent, as 14-day survey periods or occasions have also been used by other studies of wild carnivores in Sumatra, including Sunda clouded leopards [19, 56]. Moreover, because more frequent interactions between two or more carnivore species can lead to the exclusion of the smaller predator(s) [68], use of longer study occasions could help address the potentially negative impact of these interactions on sampling.

Investigations of long-term circadian activity patterns among carnivores and their prey can yield further information on coexistence and competition mechanisms among sympatric species [17, 69]. Dholes, sun bears, and tigers were all generally active during the day (diurnal) in this study (>50% of occurrences). Only Sunda clouded leopards and tigers tended not to overlap temporally with people, including our own team working in the field, suggesting a temporal avoidance of humans at all sites. The activity of sun bears and dholes exhibited temporal overlap with people and tigers, and clouded leopards had very high temporal overlap with tigers (Δ = 0.74) overall due principally to overlap at two study sites: Northeastern Rimbang Baling (Δ = 0.69) and Northwestern Rimbang Baling (Δ = 0.50). Closer to human-dominated habitats, these carnivores were more likely to increase nocturnal or crepuscular activity and were active less during the day, likely an attempt to avoid people. This suggests that spatial segregation may be more important for these species to avoid each other [2, 70].

Of course, greater spatiotemporal overlap among humans and large carnivore species may increase the potential or likelihood of conflict scenarios. In such contexts, human-carnivore conflict could be more common in intermediately disturbed areas, such as around multiple-use forests [71]. Human-bear conflicts, where sun bears attacked and mauled people causing injuries, were recently recorded in the communities around northeastern Rimbang Baling and Bukit Bungkuk. We also learned that during the course of our study, low intensity human-tiger conflict occurred across our study area; however, these incidents were often reported by communities as tigers passed through their plantations, and so may have been based more on fear than substance. Regardless, human-tiger conflict is more likely to occur closer to human villages or settlements in and around forest areas where wild prey occupancy is relatively low, livestock density is relatively high, and recent deforestation or habitat conversion has occurred [72]. Knowledge as to how overlap between people and large carnivores effects species behavior is fundamental to the development of effective wildlife conservation and management strategies, especially those aiming to achieve multi-species coexistence at a finer scale.

Our study also highlighted temporal overlap between people and several potential prey species, as well as tigers and these prey species. For example, wild boar had high temporal overlap with humans across all study sites. We inferred that it occurred because both people and wild boar were more likely to interact at the same time (i.e., hunters pursing wild boar), and because boar were widespread across the study area with higher occupancy rates relative to other potential prey species. High temporal overlap between muntjac and tigers (Δ = 0.78) in all study sites suggested the contribution of the former to the survival of the local tiger population, an important finding particularly given the low occupancy rates of sambar deer.

### Recommendations for conservation

The conservation of large carnivore populations across rapidly changing landscapes requires current and detailed information at multiple scales; yet, financial and personnel resources to gather this fundamental information are often very limited [45, 73]. Among the carnivores in our study, tigers have generally attracted the strongest global research attention; consequently, this has meant more data has been available for them [74, 75]. Alternatively, the other three “large” carnivores, *i*.*e*., dholes, Sunda clouded leopards, and Malayan sun bears, have received much less attention. Despite their ‘Vulnerable’ IUCN Redlist status [52, 76], the status and ecology of Sunda clouded leopards and Malayan sun bears are imperfectly understood. Key metrics, such as abundance and occupancy, have been challenging to obtain for Sunda clouded leopards [25]. Similarly, dholes, a canid as globally ‘Endangered’ as tigers, suffer from a similar lack of research and conservation focus in Sumatra [58]. Investigations into the population dynamics, movements, and seasonal ecology of these species and their prey are therefore incredibly important, and strongly recommended.

In addition to human presence across our study sites, our camera-traps also recorded motorbikes and vehicles in Bukit Betabuh and Tesso Nilo, which unfortunately confirms these two areas are increasingly accessible. In fact, a high density of roads now occurs around Tesso Nilo NP, a conservation area that is supposed to be managed as a key priority area for local wildlife [47]. Controlling the expansion of road construction, which can potentially have severe impacts on wildlife populations, must be a key compromise to ensure their protection and integrity [77]. With a growing human population, infrastructure on Sumatra, particularly roads, can be expected to continue expanding rapidly. Preserving remaining forests, as well as restoring habitat connectivity, is one of the major recommendations of Tesso Nilo NP conservation planning efforts [51], and this could be remedied effectively at the policy level.

For Rimbang Baling, one priority must be to ensure the connectivity of wildlife populations through a potential habitat corridor of mixed secondary forest and plantations to Bukit Tigapuluh NP [78]. Plantation concessions, including mixed palm oil and *Acacia*, may be enable wildlife movement and dispersal [46, 79]. In addition, surveys of non-core landscapes, including secondary forests, buffer zones, and wildlife corridors, are critically necessary to determine their effectiveness in achieving carnivore conservation milestones, and capacity of these populations to persist over time despite development and habitat alteration [80]. For core habitat like protected areas, we also recommend measuring the impacts of intervention and management effectiveness against important conservation standards and metrics, as such needs still represent important gaps for tigers [73], and other carnivore species as well.

Finally, large carnivore population declines are of a global concern, as they are typically precipitated by human-caused threats, including poaching, habitat loss, and depletion of prey [6], all of which can have cascading effects across ecosystems. In Lambir National Park (Malaysian Borneo), excessive hunting led to severe defaunation, which in turn instigated dramatic changes to tree recruitment dynamics [81]. Across all of Southeast Asia including Sumatra, a snaring crisis has led to extreme defaunation across the entire biogeographical region [82]. Another rapidly-growing and more recent concern for the region is the rapid emergence and expansion of disease, such as African Swine Flu, which is capable of killing large numbers of native pig species and other wildlife [83].

Lastly, our camera-traps recorded seriously injured and three legged-animals, including sun bears and bearded pigs. This is evidence that illegal hunting by people across our study sites, including the use of wire snares targeting tigers and nylon snares targeting potential prey species to address local human protein needs, across our study sites. All of this is likely causing additional non-lethal and lethal ‘by-catch’ of non-target species. To combat defaunation caused by local snaring and other forms of poaching, we recommend the expansion of, and redoubling of investment in, strategies that involve the use of local informants and an increase in the spatial coverage of patrols, both of which are currently being implemented [24, 84]. We also call for greater, more sustained levels of engagement with local rural communities about human-wildlife conflict and sustainable livelihood opportunities. We support the recommendations of other studies [84], and call for more formal or scientific evaluations of integrated protection strategies to assess their comparative efficacy and performance in protecting Sumatra’s carnivores from the various threats we have discussed throughout this study.

## Supporting information

S1 File Alternative language abstract. (DOCX)

S1 Table Habitat covariates used in modelling occupancy for this study. (DOCX)

S2 Table Pearson’s correlation among field-derived and GIS-extracted covariates. Distance to forest edges is “DistFor”; distance to big rivers is “DistRiv”; distance to roads is “DistRoad” and altitude is “Alt”. The measurement units are in meters (*m*).
(DOCX)

S3 Table Descriptions of the parameters used in the conditional two–species occupancy model. Species A is assumed to be dominant, and species B is subordinate. We assumed people and tigers as the apex predators in Sumatran ecosystems (species A), whereas the three other large carnivores and potential prey species (species B) were affected by people and tigers. With respect to predation, we used potential prey species as species A, and tigers as the “response” species (species B).
(DOCX)

S4 Table Number of independent photographs. RBNE is Rimbang Baling Northeastern; RBNW is Rimbang Baling Northwestern; RBSt is Rimbang Baling Southern; CABB is Bukit Bungkuk; HLBB is Bukit Betabuh and TNTN is Tesso Nilo; IP is number of independent events (number of photographs/30 minutes).
(DOCX)

S5 Table Independent photographs of human activities in all study sites. Independent records were photographs or “samples” that were separated by at least 30 minutes; IP is number of independent events (number of photographs/30 minutes); CTR is camera-trapping success rates (independent events/100 trap nights).
(DOCX)

S6 Table Naive occupancy and probability of occupancy (ψ) with 95% CI based on model averages for top models with ΔAICc ≤ 2: People, large carnivores, and potential prey species for 147 camera stations across all study sites. (DOCX)

S7 Table The 10 top occupancy models for people, large carnivores, and potential prey species across all study sites. We ran a total of 24 different models for each potential prey species using an AIC_c_ (Akaike information criterion), model selection framework, where the lowest AIC_c_ was the best model. We used field-derived and GIS-extracted covariates, which included “distance to forest edges” (DistFor), “distance to big rivers” (DistRiv), “distance to roads” (DistRoad), and “altitude” (Alt) or elevation.
(DOCX)

S8 Table Beta estimates (β) of the best models for people, four large carnivores, and potential prey species (based on the best models in S6 Table). These beta estimates are from the model with the lowest AIC_c_ in the model selection framework.
(DOCX)

S9 Table The top 10 two–species occupancy models of people and large carnivores from all study sites, as adapted from conditional two–species occupancy models. We ran a total of 78 different models to examine two–species occupancy. We used field-derived and GIS-extracted covariates, including “distance-to-forest edges” (DistFor), “distance-to-big rivers” (DistRiv), “distance-to-roads” (DistRoad), and “altitude” (Alt) or elevation. “A” is people, as the dominant species, and “B” are subordinate species, and number of parameters = df (degrees of freedom) for the adjusted Akaike Information Criterion (AIC_c_), where the lowest value represented the best model. We considered all models with ΔAIC ≤ 2.0 as competing models in the same information-theoretic framework.
(DOCX)

S10 Table Spatial overlap between people (dominant, species A) and large carnivores (subordinate, species B) based on model-averaged ΔAICc ≤ 2 for 147 camera-trap stations across all study sites. (DOCX)

S11 Table Spatial overlap between Sumatran tigers (dominant, species A) and three other large carnivores (subordinate, species B) based on model-averaged ΔAICc ≤ 2 for 147 camera stations across all study sites. (DOCX)

S12 Table Spatial overlap between people (dominant, species A) and potential prey species (subordinate, species B) based on model-averaged ΔAICc ≤ 2 for 147 camera stations and across all study sites. (DOCX)

S13 Table Spatial overlap between potential prey species (dominant, species A) and Sumatran tigers (subordinate, species B) based on model-averaged ΔAICc ≤ 2 for 147 camera stations across all study sites. (DOCX)

S14 Table Naive proportion of temporal activity patterns (diurnal, nocturnal and crepuscular) as indicated by the number of independent photographs, the percentage (%) of all diel activity for people, large carnivores, and potential prey species across all study sites. (DOCX)
