# Supplementary material for: Carnivores and their prey in Sumatra: Occupancy and activity in human-dominated forests
Source: PLoS One. 2022 Mar 18;17(3):e0265440. doi: 10.1371/journal.pone.0265440 (PMC8932565; doi:10.1371/journal.pone.0265440)
Supplement: S1 File — (DOCX) [file pone.0265440.s001.docx]

**S1 Alternative Language Abstract**

Memahami dampak gangguan antropogenik, dan interaksinya dengan karnivora dan mangsanya sangat penting untuk mendukung konservasi karnivora terancam, khususnya di lanskap dengan perubahan yang cepat. Berdasarkan pengambilan sampel dengan kamera penjebak yang dipasang secara sistematis di empat kawasan konservasi dan lindung di Provinsi Riau, Sumatera bagian tengah, kami menilai hunian habitat dan tumpang tindih secara spasial dan temporal antara manusia, mangsa karnivora potensial, dan empat spesies karnivora berukuran sedang atau besar yang terancam: harimau Sumatera (*Panthera tigris sumatrae*), beruang madu Malaya (*Helarctos malayanus*), ajak (*Cuon alpinus*), dan macan dahan Sunda (*Neofelis diardi*). Untuk menilai tumpang tindih spasial spesies target, kami menggunakan model hunian spesies-tunggal (*single-species occupancy*) dan menerapkan faktor interaksi spesies (*Species Interaction Factor*/ SIF) ke model hunian dua-spesies bersyarat (*conditional two-species occupancy*). Kami juga menggunakan estimasi kepadatan kernel (Kernel Density Estimation/ KDE) untuk menilai tumpang tindih temporal antar spesies. Model penggunaan habitat kami menunjukkan bahwa ketinggian (elevasi) sangat memengaruhi hunian semua karnivora besar dan spesies mangsa potensial. Kecuali macan dahan Sunda, keberadaan spesies karnivora besar berhubungan positif dengan keberadaan manusia secara spasial (SIF > 1). Selain itu, kami menampilkan bahwa beruang madu dan ajak keduanya menunjukkan tumpang tindih spasial yang tinggi dengan harimau, dan secara temporal, beruang madu menunjukkan tumpang tindih yang tinggi dengan manusia. Temuan kami berkontribusi pada peningkatan pemahaman tentang ekologi kontemporer karnivora dan mangsanya di lanskap – lanskap yang berubah dengan cepat di Asia Tenggara. Pengetahuan tersebut penting untuk konservasi dan pemulihan karnivora besar di kawasan – kawasan konservasi yang semakin didominasi oleh manusia di seluruh Sumatera, serta secara global.
