# Supplementary material for: Carnivores and their prey in Sumatra: Occupancy and activity in human-dominated forests
Source: PLoS One. 2022 Mar 18;17(3):e0265440. doi: 10.1371/journal.pone.0265440 (PMC8932565; doi:10.1371/journal.pone.0265440)
Supplement: S1 Table — (DOCX) [file pone.0265440.s002.docx]

**S2 Table. Habitat covariates used in modelling occupancy for this study.**

| **No** | **Covariate** | **Notes** |
| --- | --- | --- |
| 1. | Altitude (*m*.asl) | Recorded in the field at each station by using GPS. |
| 2. | Distance to forest edges | Using forest cover 2012 [1]. The land use monitoring was a continuation of previous monitoring data [2] and the previous map layers were used in previous carnivore studies [3,4]. Forest cover layers were generated by utilizing the best satellite images during the period of monitoring provided by Landsat USGS with resolution 30 meters. We categorized natural forests, secondary forests and remnant forests as forest cover in map layers. We integrated all forest layers by using the dissolve function in ArcGIS 10.4 [5]. Shapefile shape is polygons. We projected the forest layer map with the World Mercator which is appropriate for areas along the equator lines. |
| 3. | Distance to roads | We included all accessible roads based on Badan Informasi Geospatial or Geospatial Information Agency 2013, an official source used by Indonesia Government [6]. Shapefile shape is polylines. Roads in Indonesia are divided by several categories: collector roads, artery roads, local roads, and other roads. The map scale is 1:50,000. We projected the map with the World Mercator which is appropriate for areas along the equator lines. |
| 4. | Distance to main rivers | We only used main rivers that are readily accessible with map layers provided by Badan Informasi Geospatial or Geospatial Information Agency, an official source used by Indonesia Government [6]. The map scale is 1:50,000. Shapefile shape is polygons. We projected the map with the World Mercator which is appropriate for areas along the equator lines. |

**Reference**

1. Setiabudi S. Land use map 2012 of Sumatra. WWF-Indonesia, Jakarta, Indonesia; 2012.

2. Uryu Y, Purastuti E, Laumonier Y, Sunarto S, Setiabudi S, Budiman A, et al. Sumatra’s forests, their wildlife and the climate windows in time: 1985, 1990, 2000 and 2009. WWF-Indonesia, Jakarta, Indonesia; 2010. Available: http://awsassets.wwf.or.id/downloads/wwf_indonesia__2010__sumatran_forests_wildlife_climate_report_for_dkn___bappenas.pdf

3. Sunarto S, Kelly MJ, Parakkasi K, Klenzendorf S, Septayuda E, Kurniawan H. Tigers need cover: multi-scale occupancy study of the big cat in Sumatran forest and plantation landscapes. PLoS ONE. 2012;7: e30859. doi:10.1371/journal.pone.0030859

4. Sunarto S, Kelly MJ, Parakkasi K, Hutajulu MB. Cat coexistence in central Sumatra: ecological characteristics, spatial and temporal overlap, and implications for management. J Zool. 2015;296: 104–115. doi:10.1111/jzo.12218

5. ESRI. ArcGIS desktop release 10.4. Redlands, CA: Environmental Systems Research Institute; 2016.

6. BIG. Badan Informasi Geospasial (Geospatial Information Agency). Bogor, Indonesia; 2013.
