# Supplementary material for: Carnivores and their prey in Sumatra: Occupancy and activity in human-dominated forests
Source: PLoS One. 2022 Mar 18;17(3):e0265440. doi: 10.1371/journal.pone.0265440 (PMC8932565; doi:10.1371/journal.pone.0265440)
Supplement: S2 Table — Distance to forest edges is “DistFor”; distance to big rivers is “DistRiv”; distance to roads is “DistRoad” and altitude is “Alt”. The measurement units are in meters (m). (DOCX) [file pone.0265440.s003.docx]

**S3 Table.** **Pearson’s correlation among field-derived and GIS-extracted covariates.** Distance to forest edges is “DistFor”; distance to big rivers is “DistRiv”, distance to roads is “DistRoad” and altitude is “Alt”. The measurement units are in meters (m).

|  | **DistFor** | **DistRiv** | **DistRoad** | **Alt** |
| --- | --- | --- | --- | --- |
| **DistFor** | 1 |  |  |  |
| **DistRiv** | 0.32 | 1 |  |  |
| **DistRoad** | 0.79 | 0.38 | 1 |  |
| **Alt** | 0.50 | –0.05 | 0.57 | 1 |
