# Supplementary material for: Carnivores and their prey in Sumatra: Occupancy and activity in human-dominated forests
Source: PLoS One. 2022 Mar 18;17(3):e0265440. doi: 10.1371/journal.pone.0265440 (PMC8932565; doi:10.1371/journal.pone.0265440)
Supplement: S3 Table — Species A is assumed to be dominant, and species B is subordinate. We assumed people and tigers as the apex predators in Sumatran ecosystems (species A), whereas the three other large carnivores and potential prey species (species B) were affected by people and tigers. With respect to predation, we used potential prey species as species A, and tigers as the “response” species (species B). (DOCX) [file pone.0265440.s004.docx]

**S4 Table. Descriptions of the parameters used in the conditional two–species occupancy model.** Species A is assumed to be dominant, and species B is subordinate. We assumed people and tigers as the apex predators in Sumatra ecosystem (species A), whereas the three other large carnivores and putative prey species (species B) were affected by people and tigers. With respect to predation, we used potential prey species as species A, against tigers (species B).

| **Parameter** | **Description** |
| --- | --- |
| ψ^A^ | Probability of occupancy for species A |
| ψ^BA^ | Probability of occupancy for species B, given species A is present |
| ψ^Ba^ | Probability of occupancy for species B, given species A is absent |
| *p*^A^ | Probability of detection for species A, given species B is absent |
| *p*^B^ | Probability of detection for species B, given species A is absent |
| *r*^A^ | Probability of detection for species A, given both species are present |
| *r*^BA^ | Probability of detection for species B, given both species are present and species A is detected |
| *r*^Ba^ | Probability of detection for species B, given both species are present and species A is not detected |
