# Supplementary material for: Carnivores and their prey in Sumatra: Occupancy and activity in human-dominated forests
Source: PLoS One. 2022 Mar 18;17(3):e0265440. doi: 10.1371/journal.pone.0265440 (PMC8932565; doi:10.1371/journal.pone.0265440)
Supplement: S4 Table — RBNE is Rimbang Baling Northeastern; RBNW is Rimbang Baling Northwestern; RBSt is Rimbang Baling Southern; CABB is Bukit Bungkuk; HLBB is Bukit Betabuh and TNTN is Tesso Nilo; IP is number of independent events (number of photographs/30 minutes). (DOCX) [file pone.0265440.s005.docx]

**S5 Table. Number of independent photographs.** RBNE is Rimbang Baling Northeastern; RBNW is Rimbang Baling Northwestern; RBST is Rimbang Baling Southern; CABB is Bukit Bungkuk; HLBB is Bukit Betabuh and TNTN is Tesso Nilo; IP is number of independent events (number of photographs/ 30 minutes).

| **Species** | **RBNE** | | **RBNW** | | **RBST** | | **CABB** | | **HLBB** | | **TNTN** | | **Total** | |
| --- | --- | --- | --- | --- | --- | --- | --- | --- | --- | --- | --- | --- | --- | --- |
|  | **IP** | **%** | **IP** | **%** | **IP** | **%** | **IP** | **%** | **IP** | **%** | **IP** | **%** | **IP** | **%** |
| People | 86 | 4.73 | 84 | 4.62 | 38 | 2.09 | 6 | 0.33 | 757 | 41.64 | 847 | 46.59 | 1818 | 37.69 |
| Clouded leopard | 45 | 23.68 | 75 | 39.47 | 10 | 5.26 | 11 | 5.79 | 11 | 5.79 | 38 | 20 | 190 | 3.94 |
| Dholes | 9 | 24.32 | 10 | 27.03 | 3 | 8.11 | 8 | 21.62 | 3 | 8.11 | 4 | 10.81 | 37 | 0.77 |
| Malayan sun bears | 74 | 13.1 | 262 | 46.37 | 112 | 19.82 | 36 | 6.37 | 22 | 3.89 | 59 | 10.44 | 565 | 11.71 |
| Sumatran tigers | 11 | 16.18 | 17 | 25 | 29 | 42.65 | 1 | 1.47 | 8 | 11.76 | 2 | 2.94 | 68 | 1.41 |
| Southern red muntjac | 83 | 12.61 | 116 | 17.63 | 179 | 27.2 | 66 | 10.03 | 97 | 14.74 | 117 | 17.8 | 658 | 13.64 |
| Bearded pigs | 0 | 0 | 454 | 97.22 | 0 | 0 | 0 | 0 | 12 | 2.57 | 1 | 0.21 | 467 | 9.68 |
| Mouse deer | 91 | 24.4 | 7 | 1.88 | 102 | 27.35 | 32 | 8.58 | 46 | 12.33 | 95 | 25.47 | 373 | 7.73 |
| Sambar deer | 3 | 20 | 3 | 20 | 0 | 0 | 2 | 13.33 | 6 | 40 | 1 | 6.67 | 15 | 0.31 |
| Sumatran serow | 2 | 10 | 16 | 80 | 2 | 10 | 0 | 0 | 0 | 0 | 0 | 0 | 20 | 0.41 |
| Wild pigs | 414 | 67.65 | 16 | 2.61 | 24 | 3.92 | 21 | 3.43 | 67 | 10.95 | 70 | 11.44 | 612 | 12.69 |
