# Supplementary material for: Carnivores and their prey in Sumatra: Occupancy and activity in human-dominated forests
Source: PLoS One. 2022 Mar 18;17(3):e0265440. doi: 10.1371/journal.pone.0265440 (PMC8932565; doi:10.1371/journal.pone.0265440)
Supplement: S5 Table — Independent records were photographs or “samples” that were separated by at least 30 minutes; IP is number of independent events (number of photographs/30 minutes); CTR is camera-trapping success rates (independent events/100 trap nights). (DOCX) [file pone.0265440.s006.docx]

**S6 Table. Independent photographs of human activities in all study sites.** Independent records were independent photographs or “samples” of were separated by 30 minutes; IP is number of independent events (number of photographs/30 minutes); CTR is camera-trapping rates (independent events/100 trap nights).

| **Object** | **Rimbang Baling Northeastern** | | | **Rimbang Baling Northwestern** | | | **Rimbang Baling Southern** | | | **Bukit Bungkuk** | | | **Bukit Betabuh** | | | **Tesso Nilo** | | | **Total** | | |
| --- | --- | --- | --- | --- | --- | --- | --- | --- | --- | --- | --- | --- | --- | --- | --- | --- | --- | --- | --- | --- | --- |
|  | **IP** | **%** | **CTR** | **IP** | **%** | **CTR** | **IP** | **%** | **CTR** | **IP** | **%** | **CTR** | **IP** | **%** | **CTR** | **IP** | **%** | **CTR** | **IP** | **%** | **CTR** |
| ***Illegal activity types*** | | | | | | | | | | | | | | | | | | | | | |
| **Bird catcher** | 41 | 56.9 | 2.42 | 46 | 60.5 | 1.47 | 26 | 90 | 0.8 | 0 | 0 | 0 | 16 | 1.7 | 0.88 | 83 | 9.8 | 3.46 | 212 | 11 | 9.03 |
| **Encroacher** | 0 | 0 | 0 | 0 | 0 | 0 | 0 | 0 | 0 | 0 | 0 | 0 | 15 | 1.6 | 0.83 | 150 | 18 | 6.25 | 165 | 8.4 | 7.08 |
| **Fish** | 0 | 0 | 0 | 0 | 0 | 0 | 2 | 6.9 | 0.1 | 0 | 0 | 0 | 0 | 0 | 0 | 12 | 1.4 | 0.5 | 14 | 0.7 | 0.56 |
| **Logger** | 26 | 36.1 | 1.6 | 9 | 11.8 | 0.28 | 0 | 0 | 0 | 0 | 0 | 0 | 900 | 96 | 49.7 | 515 | 61 | 21.56 | 1450 | 74 | 73.19 |
| **NTFP** | 0 | 0 | 0 | 4 | 5.26 | 0.12 | 0 | 0 | 0 | 1 | 100 | 0.05 | 0 | 0 | 0 | 29 | 3.4 | 1.2 | 34 | 1.7 | 1.38 |
| **Poacher** | 3 | 4.17 | 0.17 | 2 | 2.63 | 0.06 | 0 | 0 | 0 | 0 | 0 | 0 | 2 | 0.2 | 0.06 | 30 | 3.5 | 1.26 | 37 | 1.9 | 1.55 |
| **Unidentified** | 2 | 2.78 | 0.11 | 15 | 19.7 | 0.47 | 1 | 3.4 | 0 | 0 | 0 | 0 | 0 | 0 | 0 | 32 | 3.8 | 1.33 | 50 | 2.5 | 1.94 |
| **Sub Total** | 72 | 100 | 4.3 | 76 | 100 | 2.4 | 29 | 100 | 0.9 | 1 | 100 | 0.05 | 933 | 100 | 51.5 | 851 | 100 | 35.56 | 1962 | 100 | 94.73 |
| ***Transportation types*** | | | | | | | | | | | | | | | | | | | | | |
| **Buffalo** | 0 | 0 | 0 | 0 | 0 | 0 | 0 | 0 | 0 | 0 | 0 | 0 | 29 | 3.1 | 1.61 | 0 | 0 | 0 | 29 | 1.5 | 1.61 |
| **Car** | 0 | 0 | 0 | 0 | 0 | 0 | 0 | 0 | 0 | 0 | 0 | 0 | 22 | 2.4 | 1.18 | 0 | 0 | 0 | 22 | 1.1 | 1.18 |
| **Elephant** | 0 | 0 | 0 | 0 | 0 | 0 | 0 | 0 | 0 | 0 | 0 | 0 | 0 | 0 | 0 | 6 | 0.7 | 0.25 | 6 | 0.3 | 0.25 |
| **Motorbike** | 0 | 0 | 0 | 0 | 0 | 0 | 0 | 0 | 0 | 0 | 0 | 0 | 234 | 25 | 13 | 689 | 79 | 28.79 | 923 | 47 | 41.79 |
| **Tractor** | 0 | 0 | 0 | 0 | 0 | 0 | 0 | 0 | 0 | 0 | 0 | 0 | 13 | 1.4 | 0.73 | 0 | 0 | 0 | 13 | 0.7 | 0.73 |
| **Truck** | 0 | 0 | 0 | 0 | 0 | 0 | 0 | 0 | 0 | 0 | 0 | 0 | 94 | 10 | 5.13 | 0 | 0 | 0 | 94 | 4.7 | 5.13 |
| **Unidentified** | 0 | 0 | 0 | 0 | 0 | 0 | 0 | 0 | 0 | 0 | 0 | 0 | 0 | 0 | 0 | 1 | 0.1 | 0.04 | 1 | 0.1 | 0.04 |
| **Walk** | 72 | 100 | 4.31 | 76 | 100 | 2.41 | 29 | 100 | 0.9 | 1 | 100 | 0.05 | 541 | 58 | 29.9 | 173 | 20 | 7.23 | 892 | 45 | 44.75 |
| **Sub Total** | 72 | 100 | 4.31 | 76 | 100 | 2.41 | 29 | 100 | 0.9 | 1 | 100 | 0.05 | 933 | 100 | 51.5 | 869 | 100 | 36.31 | 1980 | 100 | 95.48 |
