# Supplementary material for: Carnivores and their prey in Sumatra: Occupancy and activity in human-dominated forests
Source: PLoS One. 2022 Mar 18;17(3):e0265440. doi: 10.1371/journal.pone.0265440 (PMC8932565; doi:10.1371/journal.pone.0265440)
Supplement: S6 Table — (DOCX) [file pone.0265440.s007.docx]

**S7 Table. Naive occupancy and probability of occupancy (ψ) with 95% CI based on model averages for top models with ∆AICc ≤ 2: people, large carnivores, and putative prey species for 147 camera stations across all study sites.**

| **Species** | **Northeastern Rimbang Baling** | | **Northwestern Rimbang Baling** | | **Southern Rimbang Baling** | | **Bukit Bungkuk** | | **Bukit Betabuh** | | **Tesso Nilo** | | **Entire study areas** | |
| --- | --- | --- | --- | --- | --- | --- | --- | --- | --- | --- | --- | --- | --- | --- |
|  | **Naive (SD)** | **Mean ψ (95% CI)** | **Naive (SD)** | **Mean ψ (95% CI)** | **Naive (SD)** | **Mean ψ (95% CI)** | **Naive (SD)** | **Mean ψ (95% CI)** | **Naive (SD)** | **Mean ψ (95% CI)** | **Naive (SD)** | **Mean ψ (95% CI)** | **Naive (SD)** | **Mean ψ (95% CI)** |
| People | 0.75 (0.44) | 0.66  (0.51 – 0.78) | 0.63 (0.49) | 0.52  (0.34 – 0.71) | 0.50 (0.51) | 0.51  (0.33 – 0.70) | 0.10 (0.31) | 0.66  (0.52 – 0.77) | 0.70 (0.47) | 0.68  (0.55 – 0.78) | 0.88 (0.33) | 0.78  (0.63 – 0.88) | 0.60 (0.49) | 0.62  (0.46 – 0.76) |
| *Large carnivore species* | | | | | | | | | | | | | | |
| Malayan sun bear | 0.70 (0.47) | 0.73  (0.56 – 0.85) | 0.93 (0.25) | 0.86  (0.69 – 0.94) | 0.91 (0.30) | 0.86  (0.69 – 0.94) | 0.60 (0.50) | 0.73  (0.59 – 0.83) | 0.10 (0.31) | 0.71  (0.58 – 0.82) | 0.76 (0.44) | 0.58  (0.39 – 0.75) | 0.71 (0.46) | 0.76  (0.59 – 0.86) |
| Sunda Clouded leopard | 0.75 (0.44) | 0.45  (0.24 – 0.67) | 0.77 (0.43) | 0.83  (0.50 – 0.95) | 0.28 (0.46) | 0.47  (0.23 – 0.73) | 0.35 (0.50) | 0.57  (0.36 – 0.76) | 0.30 (0.47) | 0.65  (0.45 – 0.80) | 0.36 (0.50) | 0.55  (0.32 – 0.76) | 0.47 (0.50) | 0.59  (0.35 – 0.78) |
| Dhole | 0.30 (0.47) | 0.61  (0.20 – 0.90) | 0.30 (0.47) | 0.54  (0.15 – 0.84) | 0.09 (0.30) | 0.32  (0.10 – 0.68) | 0.30 (0.47) | 0.60  (0.21 – 0.88) | 0.20 (0.41) | 0.67  (0.23 – 0.92) | 0.12 (0.33) | 0.60  (0.15 – 0.92) | 0.21 (0.41) | 0.54  (0.17 – 0.85) |
| Sumatran tiger | 0.30 (0.47) | 0.36  (0.14 – 0.63) | 0.37 (0.49) | 0.61  (0.25 – 0.85) | 0.50 (0.51) | 0.70  (0.33 – 0.91) | 0.05 (0.22) | 0.33  (0.15 – 0.57) | 0.20 (0.41) | 0.28  (0.13 – 0.49) | 0.04 (0.20) | 0.14  (0.04 – 0.35) | 0.27 (0.44) | 0.43  (0.19 – 0.66) |
| *Putative prey species* | | | | | | | | | | | | | | |
| Southern red muntjac | 0.80 (0.41) | 0.91 (0.79 – 0.97) | 0.67 (0.48) | 0.68 (0.50 – 0.83) | 0.97 (0.18) | 0.91 (0.75 – 0.97) | 0.85 (0.37) | 0.91 (0.79 – 0.96) | 0.80 (0.41) | 0.88 (0.75 – 0.95) | 0.92 (0.28) | 0.94 (0.79 – 0.98) | 0.84 (0.37) | 0.86 (0.72 – 0.94) |
| Bearded pig | 0.00 (0.00) | 0.06 (0.01 – 0.20) | 0.93 (0.25) | 0.87 (0.41 – 0.97) | 0.00 (0.00) | 0.08 (0.02 – 0.30) | 0.00 (0.00) | 0.20 (0.07 – 0.42) | 0.25 (0.44) | 0.36 (0.11 – 0.60) | 0.04 (0.20) | 0.15 (0.04 – 0.40) | 0.23 (0.42) | 0.30 (0.12 – 0.50) |
| Mouse deer | 0.55 (0.51) | 0.50 (0.33 – 0.66) | 0.03 (0.18) | 0.03 (0.01 – 0.09) | 0.28 (0.46) | 0.27 (0.16 – 0.41) | 0.30 (0.47) | 0.36 (0.24 – 0.50) | 0.25 (0.44) | 0.23 (0.15 – 0.37) | 0.56 (0.51) | 0.61 (0.41 – 0.78) | 0.31 (0.47) | 0.32 (0.20 – 0.45) |
| Sambar deer | 0.10 (0.31) | 0.15 (0.04 – 0.41) | 0.07 (0.25) | 0.11 (0.02 – 0.39) | 0.00 (0.00) | 0.08 (0.02 – 0.36) | 0.10 (0.31) | 0.15 (0.05 – 0.39) | 0.15 (0.37) | 0.17 (0.05 – 0.42) | 0.04 (0.20) | 0.20 (0.05 – 0.51) | 0.07 (0.25) | 0.14 (0.04 – 0.41) |
| Sumatran serow | 0.05 (0.22) | 0.04 (0.00 – 0.63) | 0.27 (0.45) | 0.51 (0.01 – 0.82) | 0.03 (0.18) | 0.12 (0.02 – 0.59) | 0.00 (0.00) | 0.02 (0.00 – 0.36) | 0.00 (0.00) | 0.02 (0.00 – 0.32) | 0.00 (0.00) | 0.00 (0.00 – 0.39) | 0.07 (0.25) | 0.14 (0.01 – 0.54) |
| Common wild pig | 1.00 (0.00) | 0.70 (0.54 – 0.82) | 0.17 (0.38) | 0.25 (0.13 – 0.44) | 0.28 (0.46) | 0.40 (0.24 – 0.60) | 0.30 (0.47) | 0.64 (0.49 – 0.77) | 0.90 (0.31) | 0.63 (0.49 – 0.76) | 0.72 (0.46) | 0.83 (0.68 – 0.92) | 0.52 (0.50) | 0.55 (0.40 – 0.70) |
