# Supplementary material for: Carnivores and their prey in Sumatra: Occupancy and activity in human-dominated forests
Source: PLoS One. 2022 Mar 18;17(3):e0265440. doi: 10.1371/journal.pone.0265440 (PMC8932565; doi:10.1371/journal.pone.0265440)
Supplement: S7 Table — We ran a total of 24 different models for each potential prey species using an AICc (Akaike information criterion), model selection framework, where the lowest AICc was the best model. We used field-derived and GIS-extracted covariates, which included “distance to forest edges” (DistFor), “distance to big rivers” (DistRiv), “distance to roads” (DistRoad), and “altitude” (Alt) or elevation. (DOCX) [file pone.0265440.s008.docx]

**S8 Table. The 10 top models of people, large carnivores, and putative prey species occupancy from all study sites**. We ran a total of 24 different models for people, large carnivores, and putative prey species. AIC_c_ is Akaike information criterion, where the lowest AIC_c_ was the best model. We used covariate field-derived and GIS-extracted which include distance of forest edges (DistFor), distance to big rivers (DistRiv), distance to roads (DistRoad), and altitude (Alt).

| **Model** | **ψ** | ***p*** | **df** | **AICc** | **∆AICc** | **ModelLik** | ***w_i_*** |
| --- | --- | --- | --- | --- | --- | --- | --- |
| a) People | | | | | | | |
| peop16 | (Alt) | (Effort) | 4 | 1079.14 | 0.00 | 1.00 | 0.17 |
| peop23 | (DistRoad+Alt) | (Effort) | 5 | 1079.47 | 0.34 | 0.85 | 0.14 |
| peop21 | (DistRiv+Alt) | (Effort) | 5 | 1079.49 | 0.35 | 0.84 | 0.14 |
| peop15 | (DistRoad) | (Effort) | 4 | 1079.81 | 0.67 | 0.72 | 0.12 |
| peop19 | (DistFor+DistRiv+Alt) | (Effort) | 6 | 1080.36 | 1.22 | 0.54 | 0.09 |
| peop18 | (DistFor+Alt) | (Effort) | 5 | 1081.01 | 1.87 | 0.39 | 0.07 |
| peop22 | (DistRiv+DistRoad+Alt) | (Effort) | 6 | 1081.05 | 1.91 | 0.39 | 0.06 |
| peop20 | (DistRiv+DistRoad) | (Effort) | 5 | 1081.93 | 2.79 | 0.25 | 0.04 |
| peop09 | (DistRiv+Alt) | (.) | 4 | 1082.56 | 3.42 | 0.18 | 0.03 |
| peop04 | (Alt) | (.) | 3 | 1082.58 | 3.45 | 0.18 | 0.03 |
| b) Malayan sun bear (*Helarctos malayanus*) | | | | | | | |
| bear16 | (Alt) | (Effort) | 4 | 1239.35 | 0.00 | 1.00 | 0.21 |
| bear21 | (DistRiv+Alt) | (Effort) | 5 | 1239.42 | 0.07 | 0.96 | 0.21 |
| bear18 | (DistFor+Alt) | (Effort) | 5 | 1240.77 | 1.42 | 0.49 | 0.11 |
| bear23 | (DistRoad+Alt) | (Effort) | 5 | 1241.06 | 1.72 | 0.42 | 0.09 |
| bear19 | (DistFor+DistRiv+Alt) | (Effort) | 6 | 1241.34 | 2.00 | 0.37 | 0.08 |
| bear22 | (DistRiv+DistRoad+Alt) | (Effort) | 6 | 1241.59 | 2.25 | 0.33 | 0.07 |
| bear15 | (DistRoad) | (Effort) | 4 | 1242.16 | 2.81 | 0.25 | 0.05 |
| bear13 | (DistFor) | (Effort) | 4 | 1242.73 | 3.38 | 0.18 | 0.04 |
| bear20 | (DistRiv+DistRoad) | (Effort) | 5 | 1243.89 | 4.54 | 0.10 | 0.02 |
| bear09 | (DistRiv+Alt) | (.) | 4 | 1244.02 | 4.68 | 0.10 | 0.02 |
| c) Sundaland clouded leopard (*Neofelis diardi*) | | | | | | | |
| clouded09 | (DistRiv+Alt) | (.) | 4 | 773.43 | 0.00 | 1.00 | 0.18 |
| clouded10 | (DistRiv+DistRoad+Alt) | (.) | 5 | 773.70 | 0.27 | 0.88 | 0.16 |
| clouded07 | (DistFor+DistRiv+Alt) | (.) | 5 | 774.41 | 0.97 | 0.62 | 0.11 |
| clouded02 | (DistRiv) | (.) | 3 | 774.64 | 1.21 | 0.55 | 0.10 |
| clouded11 | (DistRoad+Alt) | (.) | 4 | 775.32 | 1.89 | 0.39 | 0.07 |
| clouded21 | (DistRiv+Alt) | (Effort) | 5 | 775.57 | 2.14 | 0.34 | 0.06 |
| clouded22 | (DistRiv+DistRoad+Alt) | (Effort) | 6 | 775.84 | 2.40 | 0.30 | 0.06 |
| clouded08 | (DistRiv+DistRoad) | (.) | 4 | 776.47 | 3.03 | 0.22 | 0.04 |
| clouded05 | (DistFor+DistRiv) | (.) | 4 | 776.48 | 3.05 | 0.22 | 0.04 |
| clouded19 | (DistFor+DistRiv+Alt) | (Effort) | 6 | 776.58 | 3.15 | 0.21 | 0.04 |
| d) Dhole (*Cuon alpinus*) | | | | | | | |
| dhole01 | (DistFor) | (.) | 3 | 332.29 | 0.00 | 1.00 | 0.18 |
| dhole06 | (DistFor+Alt) | (.) | 4 | 332.34 | 0.05 | 0.97 | 0.17 |
| dhole13 | (DistFor) | (Effort) | 4 | 334.01 | 1.72 | 0.42 | 0.08 |
| dhole07 | (DistFor+DistRiv+Alt) | (.) | 5 | 334.13 | 1.84 | 0.40 | 0.07 |
| dhole03 | (DistRoad) | (.) | 3 | 334.36 | 2.06 | 0.36 | 0.06 |
| dhole05 | (DistFor+DistRiv) | (.) | 4 | 334.40 | 2.11 | 0.35 | 0.06 |
| dhole18 | (DistFor+Alt) | (Effort) | 5 | 334.44 | 2.15 | 0.34 | 0.06 |
| dhole00 | (.) | (.) | 2 | 334.64 | 2.35 | 0.31 | 0.06 |
| dhole11 | (DistRoad+Alt) | (.) | 4 | 335.53 | 3.24 | 0.20 | 0.04 |
| dhole17 | (DistFor+DistRiv) | (Effort) | 5 | 336.15 | 3.86 | 0.15 | 0.03 |
| e) Sumatran tiger (*Panthera tigris sumatrae*) | | | | | | | |
| tiger06 | (DistFor+Alt) | (.) | 4 | 435.88 | 0.00 | 1.00 | 0.15 |
| tiger09 | (DistRiv+Alt) | (.) | 4 | 436.19 | 0.31 | 0.86 | 0.13 |
| tiger07 | (DistFor+DistRiv+Alt) | (.) | 5 | 436.61 | 0.73 | 0.69 | 0.10 |
| tiger11 | (DistRoad+Alt) | (.) | 4 | 437.16 | 1.28 | 0.53 | 0.08 |
| tiger21 | (DistRiv+Alt) | (Effort) | 5 | 437.66 | 1.78 | 0.41 | 0.06 |
| tiger10 | (DistRiv+DistRoad+Alt) | (.) | 5 | 437.81 | 1.93 | 0.38 | 0.06 |
| tiger18 | (DistFor+Alt) | (Effort) | 5 | 437.99 | 2.11 | 0.35 | 0.05 |
| tiger04 | (Alt) | (.) | 3 | 437.99 | 2.11 | 0.35 | 0.05 |
| tiger19 | (DistFor+DistRiv+Alt) | (Effort) | 6 | 438.57 | 2.69 | 0.26 | 0.04 |
| tiger01 | (DistFor) | (.) | 3 | 438.28 | 2.40 | 0.30 | 0.05 |
| f)    Southern red muntjac (*Muntiacus muntjak*) | | | | | | | |
| bark11 | (DistRoad+Alt) | (.) | 4 | 1393.16 | 0.00 | 1.00 | 0.24 |
| bark06 | (DistFor+Alt) | (.) | 4 | 1394.10 | 0.94 | 0.63 | 0.15 |
| bark23 | (DistRoad+Alt) | (Effort) | 5 | 1394.45 | 1.29 | 0.53 | 0.13 |
| bark10 | (DistRiv+DistRoad+Alt) | (.) | 5 | 1395.06 | 1.90 | 0.39 | 0.09 |
| bark18 | (DistFor+Alt) | (Effort) | 5 | 1395.30 | 2.14 | 0.34 | 0.08 |
| bark07 | (DistFor+DistRiv+Alt) | (.) | 5 | 1395.50 | 2.34 | 0.31 | 0.08 |
| bark09 | (DistRiv+Alt) | (.) | 4 | 1396.23 | 3.08 | 0.22 | 0.05 |
| bark22 | (DistRiv+DistRoad+Alt) | (Effort) | 6 | 1396.34 | 3.18 | 0.20 | 0.05 |
| bark19 | (DistFor+DistRiv+Alt) | (Effort) | 6 | 1396.69 | 3.54 | 0.17 | 0.04 |
| bark04 | (Alt) | (.) | 3 | 1396.86 | 3.71 | 0.16 | 0.04 |
| g)    Bearded pigs (*Sus barbatus*) | | | | | | | |
| beard21 | (DistRiv+Alt) | (Effort) | 5 | 456.92 | 0.00 | 1.00 | 0.50 |
| beard22 | (DistRiv+DistRoad+Alt) | (Effort) | 6 | 458.27 | 1.35 | 0.51 | 0.25 |
| beard19 | (DistFor+DistRiv+Alt) | (Effort) | 6 | 458.70 | 1.78 | 0.41 | 0.21 |
| beard20 | (DistRiv+DistRoad) | (Effort) | 5 | 462.39 | 5.47 | 0.07 | 0.03 |
| beard14 | (DistRiv) | (Effort) | 4 | 465.42 | 8.49 | 0.01 | 0.01 |
| beard17 | (DistFor+DistRiv) | (Effort) | 5 | 467.46 | 10.54 | 0.01 | 0.00 |
| beard09 | (DistRiv+Alt) | (.) | 4 | 483.70 | 26.78 | 0.00 | 0.00 |
| beard10 | (DistRiv+DistRoad+Alt) | (.) | 5 | 483.95 | 27.03 | 0.00 | 0.00 |
| beard07 | (DistFor+DistRiv+Alt) | (.) | 5 | 484.50 | 27.58 | 0.00 | 0.00 |
| beard18 | (DistFor+Alt) | (Effort) | 5 | 492.97 | 36.04 | 0.00 | 0.00 |
| h)     Mouse deer (*Trangulus spp*) | | | | | | | |
| mous10 | (DistRiv+DistRoad+Alt) | (.) | 5 | 605.66 | 0.00 | 1.00 | 0.45 |
| mous11 | (DistRoad+Alt) | (.) | 4 | 606.78 | 1.13 | 0.57 | 0.26 |
| mous22 | (DistRiv+DistRoad+Alt) | (Effort) | 6 | 607.59 | 1.93 | 0.38 | 0.17 |
| mous23 | (DistRoad+Alt) | (Effort) | 5 | 608.76 | 3.10 | 0.21 | 0.10 |
| mous09 | (DistRiv+Alt) | (.) | 4 | 613.92 | 8.26 | 0.02 | 0.01 |
| mous07 | (DistFor+DistRiv+Alt) | (.) | 5 | 614.12 | 8.46 | 0.02 | 0.01 |
| mous21 | (DistRiv+Alt) | (Effort) | 5 | 615.83 | 10.17 | 0.01 | 0.00 |
| mous19 | (DistFor+DistRiv+Alt) | (Effort) | 6 | 616.02 | 10.36 | 0.01 | 0.00 |
| mous06 | (DistFor+Alt) | (.) | 4 | 618.76 | 13.10 | 0.00 | 0.00 |
| mous18 | (DistFor+Alt) | (Effort) | 5 | 620.70 | 15.04 | 0.00 | 0.00 |
| i)    Sambar deer (*Rusa unicolor*) | | | | | | | |
| Samb01 | (DistFor) | (.) | 3 | 137.86 | 0.00 | 1.00 | 0.12 |
| Samb00 | (.) | (.) | 2 | 138.10 | 0.24 | 0.89 | 0.10 |
| Samb04 | (Alt) | (.) | 3 | 138.32 | 0.46 | 0.80 | 0.09 |
| Samb03 | (DistRoad) | (.) | 3 | 138.42 | 0.56 | 0.76 | 0.09 |
| Samb13 | (.) | (Effort) | 4 | 139.35 | 1.49 | 0.48 | 0.05 |
| Samb15 | (DistRiv) | (Effort) | 4 | 139.39 | 1.52 | 0.47 | 0.05 |
| Samb06 | (DistFor+Alt) | (.) | 4 | 139.54 | 1.67 | 0.43 | 0.05 |
| Samb16 | (DistRoad) | (Effort) | 4 | 139.55 | 1.68 | 0.43 | 0.05 |
| Samb02 | (DistRiv) | (.) | 3 | 139.72 | 1.86 | 0.40 | 0.05 |
| Samb09 | (DistRiv+Alt) | (.) | 4 | 139.81 | 1.95 | 0.38 | 0.04 |
| j)     Sumatran serows (*Capricornis sumatraensis*) | | | | | | | |
| sero04 | (Alt) | (.) | 3 | 122.73 | 0.00 | 1.00 | 0.25 |
| sero09 | (DistRiv+Alt) | (.) | 4 | 123.01 | 0.28 | 0.87 | 0.22 |
| sero06 | (DistFor+Alt) | (.) | 4 | 123.76 | 1.03 | 0.60 | 0.15 |
| sero11 | (DistRoad+Alt) | (.) | 4 | 124.16 | 1.43 | 0.49 | 0.12 |
| sero21 | (DistRiv+Alt) | (Effort) | 5 | 124.70 | 1.97 | 0.37 | 0.09 |
| sero16 | (Alt) | (Effort) | 4 | 124.84 | 2.11 | 0.35 | 0.09 |
| sero18 | (DistFor+Alt) | (Effort) | 5 | 125.90 | 3.17 | 0.21 | 0.05 |
| sero23 | (DistRoad+Alt) | (Effort) | 5 | 126.29 | 3.57 | 0.17 | 0.04 |
| sero12 | (.) | (Effort) | 3 | 137.99 | 15.27 | 0.00 | 0.00 |
| sero14 | (DistRiv) | (Effort) | 4 | 138.67 | 15.95 | 0.00 | 0.00 |
| k)     Common wild pigs (*Sus scrofa*) | | | | | | | |
| pigs16 | (Alt) | (Effort) | 4 | 898.30 | 0.00 | 1.00 | 0.26 |
| pigs22 | (DistRiv+DistRoad+Alt) | (Effort) | 6 | 899.07 | 0.78 | 0.68 | 0.18 |
| pigs21 | (DistRiv+Alt) | (Effort) | 5 | 899.25 | 0.95 | 0.62 | 0.16 |
| pigs19 | (DistFor+DistRiv+Alt) | (Effort) | 6 | 899.50 | 1.20 | 0.55 | 0.14 |
| pigs23 | (DistRoad+Alt) | (Effort) | 5 | 899.82 | 1.52 | 0.47 | 0.12 |
| pigs18 | (DistFor+Alt) | (Effort) | 5 | 899.85 | 1.56 | 0.46 | 0.12 |
| pigs04 | (Alt) | (.) | 3 | 907.24 | 8.95 | 0.01 | 0.00 |
| pigs20 | (DistRiv+DistRoad) | (Effort) | 5 | 907.48 | 9.19 | 0.01 | 0.00 |
| pigs10 | (DistRiv+DistRoad+Alt) | (.) | 5 | 907.76 | 9.47 | 0.01 | 0.00 |
| pigs07 | (DistFor+DistRiv+Alt) | (.) | 5 | 907.98 | 9.68 | 0.01 | 0.00 |
