# Supplementary material for: Carnivores and their prey in Sumatra: Occupancy and activity in human-dominated forests
Source: PLoS One. 2022 Mar 18;17(3):e0265440. doi: 10.1371/journal.pone.0265440 (PMC8932565; doi:10.1371/journal.pone.0265440)
Supplement: S8 Table — These beta estimates are from the model with the lowest AICc in the model selection framework. (DOCX) [file pone.0265440.s009.docx]

**S9 Table. Beta estimates (β) of the best models for people, four large carnivores, and putative prey species (based on the best models in S6 Table).** These beta estimates are from the lowest AIC_c_ in the model selection framework.

| **Species** | **Beta (95% CI)** | | | |
| --- | --- | --- | --- | --- |
|  | **DistFor** | **DistRiv** | **DistRoad** | **Alt** |
| **People** | - | - | - | -0.51 ((-0.88) - (-0.14))* |
| **Clouded leopard** | - | -0.61 ((-1.07) - (-0.15))* | - | 0.47 ((-0.08) - 1.02) |
| **Dhole** | -0.79 ((-1.74)) - 0.15) | - | - | - |
| **Malayan sun bear** | - | - | - | 0.69 (0.19 - 1.19)* |
| **Sumatran tiger** | 0.64 (0.00 - 1.27) | - | - | 0.63 ((-0.06) - 1.32) |
| **Southern red muntjac** | - | - | 0.92 (0.08 - 1.77)* | -1.28 ((-1.95) - (-0.61))* |
| **Bearded pig** | - | -2.09 ((-3.00) - (-1.19))* |  | 1.54 (0.64 - 2.45)* |
| **Mouse deer** | - | 0.50 ((-0.05) - 1.06) | 1.25 (0.41 - 2.09)* | -3.37 ((-4.71) - (-2.03))* |
| **Sambar deer** | -0.61 ((-1.47) - 0.24) | - | - | - |
| **Sumatran serow** | - | - | - | 2.46 (0.56 - 4.35)* |
| **Common wild pig** | - | - | - | -1.17 ((-1.65) - (-0.69))* |

*Strong robust relationship indicated by 95% CI is not overlapping with 0.
