# Supplementary material for: Carnivores and their prey in Sumatra: Occupancy and activity in human-dominated forests
Source: PLoS One. 2022 Mar 18;17(3):e0265440. doi: 10.1371/journal.pone.0265440 (PMC8932565; doi:10.1371/journal.pone.0265440)
Supplement: S9 Table — We ran a total of 78 different models to examine two–species occupancy. We used field-derived and GIS-extracted covariates, including “distance-to-forest edges” (DistFor), “distance-to-big rivers” (DistRiv), “distance-to-roads” (DistRoad), and “altitude” (Alt) or elevation. “A” is people, as the dominant species, and “B” are subordinate species, and number of parameters = df (degrees of freedom) for the adjusted Akaike Information Criterion (AICc), where the lowest value represented the best model. We considered all models with ΔAIC ≤ 2.0 as competing models in the same information-theoretic framework. (DOCX) [file pone.0265440.s010.docx]

**S10 Table.** **The 10 top two–species occupancy models of people and large carnivores from all study sites****, as adapted from conditional two–species occupancy models.** We ran a total of 78 different models to examine two–species occupancy. We used field-derived and GIS-extracted covariates, including distance-to-forest edges (DistFor), distance-to-big rivers (DistRiv), distance-to-roads (DistRoad), and altitude (Alt). “A” is people, as the dominant species, and “B” are subordinate species, and number of parameters = df (degrees of freedom) for adjusted Akaike Information Criterion (AIC_c_), with the lowest value as the best model. We considered all models with ΔAIC ≤ 2.0 as competing models in the same information-theoretic framework.

| **Model** | **ψ^A^** | **ψ^BA^** | **ψ^Ba^** | ***p*A** | ***p*B** | ***r*A** | ***r*Ba** | ***r*BA** | **df** | **AICc** | **∆AICc** | **ModelLik** | ***w_i_*** |
| --- | --- | --- | --- | --- | --- | --- | --- | --- | --- | --- | --- | --- | --- |
| **a) People + Sundaland Clouded Leopard** | | | | | | | | | | | | | |
| **MDPC10** | Alt | (DistRiv+Alt) | (.) | (Effort) | (.) | (Effort) | (.) | (.) | 13 | 1829.045 | 0.000 | 1.000 | 0.609 |
| **MDPC11** | Alt | (DistRiv+Alt) | (Alt) | (Effort) | (.) | (Effort) | (.) | (.) | 14 | 1831.019 | 1.973 | 0.373 | 0.227 |
| **MDPC02** | (Alt) | (.) | (.) | (Effort) | (.) | (Effort) | (.) | (.) | 11 | 1833.562 | 4.517 | 0.105 | 0.064 |
| **MDPC07** | (Alt) | (Alt) | (.) | (Effort) | (.) | (Effort) | (.) | (.) | 12 | 1834.135 | 5.090 | 0.078 | 0.048 |
| **MDPC08** | (Alt) | (.) | (Alt) | (Effort) | (.) | (Effort) | (.) | (.) | 12 | 1835.658 | 6.613 | 0.037 | 0.022 |
| **b) People + Malayan sun bears** | | | | | | | | | | | | | |
| **MDPB05** | (Alt) | (Alt) | (Alt) | (Effort) | (Effort) | (Effort) | (Effort) | (Effort) | 16 | 2272.220 | 0.000 | 1.000 | 0.584 |
| **MDPB08** | (Alt) | (.) | (Alt) | (Effort) | (Effort) | (Effort) | (Effort) | (Effort) | 15 | 2273.575 | 1.355 | 0.508 | 0.296 |
| **MDPB12** | (.) | (DistRiv+Alt) | (Alt) | (Effort) | (Effort) | (Effort) | (Effort) | (Effort) | 16 | 2276.701 | 4.481 | 0.106 | 0.062 |
| **MDPB06** | (.) | (Alt) | (Alt) | (Effort) | (Effort) | (Effort) | (Effort) | (Effort) | 15 | 2278.138 | 5.919 | 0.052 | 0.030 |
| **MDPB04** | (.) | (.) | (Alt) | (Effort) | (Effort) | (Effort) | (Effort) | (Effort) | 14 | 2279.917 | 7.697 | 0.021 | 0.012 |
| **c) People + dholes** | | | | | | | | | | | | | |
| **MDPD07** | (Alt) | (Alt) | (.) | Effort | (.) | Effort | (.) | (.) | 12 | 1328.497 | 0.000 | 1.000 | 0.443 |
| **MDPD10** | (Alt) | (DistFor+Alt) | (.) | Effort | (.) | Effort | (.) | (.) | 13 | 1330.303 | 1.806 | 0.405 | 0.179 |
| **MDPD05** | (Alt) | (Alt) | (Alt) | Effort | (.) | Effort | (.) | (.) | 13 | 1330.474 | 1.977 | 0.372 | 0.165 |
| **MDPD03** | (.) | (Alt) | (.) | Effort | (.) | Effort | (.) | (.) | 11 | 1332.069 | 3.572 | 0.168 | 0.074 |
| **MDPD11** | (Alt) | (DistFor+Alt) | (Alt) | Effort | (.) | Effort | (.) | (.) | 14 | 1332.276 | 3.779 | 0.151 | 0.067 |
| **d) People + Sumatran tigers** | | | | | | | | | | | | | |
| **MDPT05** | (Alt) | (Alt) | (Alt) | (Effort) | (.) | (Effort) | (.) | (.) | 13 | 1360.488 | 0.000 | 1.000 | 0.499 |
| **MDPT11** | (Alt) | (DistFor+Alt) | (Alt) | (Effort) | (.) | (Effort) | (.) | (.) | 14 | 1360.831 | 0.343 | 0.842 | 0.420 |
| **MDPT06** | (.) | (Alt) | (Alt) | (Effort) | (.) | (Effort) | (.) | (.) | 12 | 1365.861 | 5.373 | 0.068 | 0.034 |
| **MDPT12** | (.) | (DistFor+Alt) | (Alt) | (Effort) | (.) | (Effort) | (.) | (.) | 13 | 1366.163 | 5.675 | 0.059 | 0.029 |
| **MDPT07** | (Alt) | (Alt) | (.) | (Effort) | (.) | (Effort) | (.) | (.) | 12 | 1368.332 | 7.844 | 0.020 | 0.010 |
| **e) Sumaran tigers + Sundaland clouded leopards** | | | | | | | | | | | | | |
| **MDTC45** | DistFor | DistFor+DistRiv+Alt | Alt | (.) | (Effort) | (.) | (Effort) | (.) | 13 | 1199.813 | 0.000 | 1.000 | 0.179 |
| **MDTC46** | Alt | DistFor+DistRiv+Alt | DistFor | (.) | (Effort) | (.) | (Effort) | (.) | 13 | 1200.444 | 0.630 | 0.730 | 0.131 |
| **MDTC29** | DistFor | DistRiv+Alt | Alt | (.) | (Effort) | (.) | (Effort) | (.) | 12 | 1200.830 | 1.017 | 0.602 | 0.108 |
| **MDTC30** | Alt | DistRiv+Alt | DistFor | (.) | (Effort) | (.) | (Effort) | (.) | 12 | 1201.448 | 1.634 | 0.442 | 0.079 |
| **MDTC47** | DistFor+Alt | DistFor+DistRiv+Alt | 1 | (.) | (Effort) | (.) | (Effort) | (.) | 13 | 1201.937 | 2.123 | 0.346 | 0.062 |
| **f) Sumaran tigers + Malayan sun bears** | | | | | | | | | | | | | |
| **MDTB43** | (Alt) | (DistFor+DistRiv+Alt) | (.) | (.) | (.) | (.) | (.) | (.) | 15 | 1660.180 | 0.000 | 1.000 | 0.744 |
| **MDTB47** | (DistFor+Alt) | (DistFor+DistRiv+Alt) | (.) | (.) | (.) | (.) | (.) | (.) | 16 | 1663.846 | 3.666 | 0.160 | 0.119 |
| **MDTB23** | (.) | (DistRiv+Alt) | (.) | (.) | (.) | (.) | (.) | (.) | 13 | 1666.920 | 6.740 | 0.034 | 0.026 |
| **MDTB16** | (DistFor+Alt) | (.) | (.) | (.) | (.) | (.) | (.) | (.) | 13 | 1667.725 | 7.546 | 0.023 | 0.017 |
| **MDTB22** | (DistFor+Alt) | (.) | (DistFor+Alt) | (.) | (.) | (.) | (.) | (.) | 15 | 1668.249 | 8.070 | 0.018 | 0.013 |
| **g) Sumatran tigers + dholes** | | | | | | | | | | | | | |
| **MDTD21** | (DistFor+Alt) | (.) | (DistFor+Alt) | (.) | (.) | (.) | (.) | (.) | 12 | 747.127 | 0.000 | 1.000 | 0.257 |
| **MDTD27** | (Alt) | (DistFor+Alt) | (DistFor) | (.) | (.) | (.) | (.) | (.) | 12 | 748.330 | 1.203 | 0.548 | 0.141 |
| **MDTD15** | (DistFor+Alt) | (.) | (.) | (.) | (.) | (.) | (.) | (.) | 10 | 749.030 | 1.903 | 0.386 | 0.099 |
| **MDTD18** | (DistFor+Alt) | (DistFor+Alt) | (DistFor+Alt) | (.) | (.) | (.) | (.) | (.) | 14 | 749.164 | 2.037 | 0.361 | 0.093 |
| **MDTD19** | (DistFor+Alt) | (DistFor+Alt) | (.) | (.) | (.) | (.) | (.) | (.) | 12 | 749.878 | 2.751 | 0.253 | 0.065 |
