# Supplementary material for: Carnivores and their prey in Sumatra: Occupancy and activity in human-dominated forests
Source: PLoS One. 2022 Mar 18;17(3):e0265440. doi: 10.1371/journal.pone.0265440 (PMC8932565; doi:10.1371/journal.pone.0265440)
Supplement: S10 Table — (DOCX) [file pone.0265440.s011.docx]

**S11 Table. Spatial overlap between people (dominant, species A) and large carnivores (subordinate, species B) based on model-averaged ∆AICc ≤ 2 for 147 camera-trap stations across all study sites.** ψ^Ba^ is the probability of occupancy for species B, given species A is absent; ψ^BA^ is the probability of occupancy for species B, given species A is present; SIF is a species interaction factor where SIF = 1 indicates two species occurred independently of each other, an SIF >1 indicates overlap, whereas an SIF <1 suggests co-occurrence is less likely. A strong SIF is indicated by 95% CI not overlapping with 1; RBNE, Northeastern Bukit Rimbang Bukit Baling; RBNW, Northwestern Bukit Rimbang Bukit Baling; RBST, Southern Bukit Rimbang Bukit Baling; CABB, Bukit Bungkuk; HLBB, Bukit Betabuh; TNTN, Tesso Nilo; All, “All study sites”.

| **Studi area** | **Naive spatially overlap (SD)** | **Mean PsiBa (95% CI)** | **Mean PsiBA (95% CI)** | **Mean SIF (95% CI)** |
| --- | --- | --- | --- | --- |
| People and Malayan sun bear | | | | |
| RBNE | 0.55 (0.51) | 0.48 (0.26 - 0.74) | 0.87 (0.73 - 0.94) | 1.14 (1.00 - 1.41) |
| RBNW | 0.60 (0.50) | 0.88 (0.67 - 0.95) | 0.96 (0.80 - 0.99) | 1.04 (0.92 - 1.20) |
| RBST | 0.47 (0.51) | 0.79 (0.58 - 0.90) | 0.93 (0.80 - 0.98) | 1.07 (0.94 - 1.24) |
| CABB | 0.05 (0.22) | 0.54 (0.32 - 0.76) | 0.88 (0.76 - 0.95) | 1.13 (1.01 - 1.28) |
| HLBB | 0.05 (0.22) | 0.55 (0.33 - 0.76) | 0.89 (0.77 - 0.95) | 1.12 (1.03 - 1.24) |
| TNTN | 0.68 (0.48) | 0.23 (0.05 - 0.63) | 0.80 (0.61 - 0.91) | 1.16 (1.14 - 1.18) |
| All | 0.43 (0.50) | 0.61 (0.40 - 0.81) | 0.89 (0.75 - 0.96) | 1.12 (0.92 - 1.58) |
| People and Sunda clouded leopard | | | | |
| RBNE | 0.60 (0.50) | 0.91 (0.00 - 1.00) | 0.47 (0.31 - 0.65) | 0.87 (0.67 - 1.01) |
| RBNW | 0.50 (0.51) | 1.00 (0.00 - 1.00) | 0.65 (0.41 - 0.84) | 0.81 (0.67 - 0.95) |
| RBST | 0.06 (0.25) | 0.99 (0.00 - 1.00) | 0.40 (0.23 - 0.60) | 0.68 (0.43 - 0.88) |
| CABB | 0.05 (0.22) | 0.94 (0.00 - 1.00) | 0.59 (0.43 - 0.72) | 0.90 (0.80 - 0.97) |
| HLBB | 0.25 (0.44) | 0.95 (0.00 - 1.00) | 0.65 (0.49 - 0.78) | 0.92 (0.85 - 0.97) |
| TNTN | 0.32 (0.48) | 0.72 (0.00 - 1.00) | 0.68 (0.46 - 0.84) | 0.99 (0.96 - 1.02) |
| All | 0.29 (0.46) | 0.92 (0.00 - 1.00) | 0.57 (0.38 - 0.74) | 0.87 (0.59 - 1.04) |
| People and dholes | | | | |
| RBNE | 0.25 (0.44) | 0.24 (0.03 - 0.75) | 0.77 (0.62 - 0.87) | 1.21 (1.08 - 1.38) |
| RBNW | 0.17 (0.38) | 0.26 (0.04 - 0.75) | 0.98 (0.90 - 0.99) | 1.48 (1.20 - 1.96) |
| RBST | 0.09 (0.30) | 0.25 (0.05 - 0.71) | 0.95 (0.85 - 0.98) | 1.36 (1.20 - 1.60) |
| CABB | 0.05 (0.22) | 0.24 (0.04 - 0.73) | 0.83 (0.69 - 0.91) | 1.23 (1.14 - 1.33) |
| HLBB | 0.20 (0.41) | 0.24 (0.04 - 0.73) | 0.85 (0.71 - 0.92) | 1.23 (1.16 - 1.31) |
| TNTN | 0.12 (0.33) | 0.23 (0.02 - 0.81) | 0.49 (0.30 - 0.69) | 1.11 (1.09 - 1.12) |
| All | 0.14 (0.35) | 0.25 (0.04 - 0.74) | 0.82 (0.69 - 0.90) | 1.27 (1.05 - 1.61) |
| People and Sumatran tigers | | | | |
| RBNE | 0.25 (0.44) | 0.01 (0.00 - 0.99) | 0.68 (0.50 - 0.82) | 1.32 (1.10 - 1.65) |
| RBNW | 0.27 (0.45) | 0.26 (0.03 - 0.95) | 0.97 (0.87 - 0.99) | 1.51 (0.97 - 4.52) |
| RBST | 0.50 (0.51) | 0.02 (0.00 - 0.95) | 0.95 (0.79 - 0.99) | 1.57 (1.25 - 2.12) |
| CABB | 0.00 (0.00) | 0.00 (0.00 - 0.99) | 0.76 (0.56 - 0.89) | 1.34 (1.19 - 1.53) |
| HLBB | 0.20 (0.41) | 0.00 (0.00 - 0.99) | 0.76 (0.56 - 0.89) | 1.35 (1.22 - 1.50) |
| TNTN | 0.04 (0.20) | 0.00 (0.00 - 1.00) | 0.37 (0.19 - 0.60) | 1.20 (1.18 - 1.22) |
| All | 0.23 (0.42) | 0.06 (0.01 - 0.97) | 0.77 (0.60 - 0.87) | 1.39 (1.01 - 2.73) |
