# Supplementary material for: Carnivores and their prey in Sumatra: Occupancy and activity in human-dominated forests
Source: PLoS One. 2022 Mar 18;17(3):e0265440. doi: 10.1371/journal.pone.0265440 (PMC8932565; doi:10.1371/journal.pone.0265440)
Supplement: S11 Table — (DOCX) [file pone.0265440.s012.docx]

**S12 Table. Spatial overlap Sumatran tigers (dominant, species A) and three other large carnivores (subordinate, species B) based on model-averaged ∆AICc ≤ 2 for 147 camera stations across all study sites.** ψ^Ba^ is the probability of occupancy for species B, given species A is absent; ψ^BA^ is the probability of occupancy for species B, given species A is present; SIF is a species interaction factor where SIF = 1 indicates two species occurred independently of each other, an SIF >1 indicates overlap, whereas an SIF <1 suggests co-occurrence is less likely. A strong SIF is indicated by 95% CI not overlapping with 1; RBNE, Northeastern Bukit Rimbang Bukit Baling; RBNW, Northwestern Bukit Rimbang Bukit Baling; RBSt, Southern Bukit Rimbang Bukit Baling; CABB, Bukit Bungkuk; HLBB, Bukit Betabuh; TNTN, Tesso Nilo; All, “All study sites”.

| **Study area** | **Naive spatially overlap (SD)** | **Mean PsiBa (95% CI)** | **Mean PsiBA (95% CI)** | **Mean SIF (95% CI)** |
| --- | --- | --- | --- | --- |
| Sumatran tigers and Malayan sun bears | | | | |
| RBNE | 0.25 (0.44) | 0.65 (0.45 - 0.81) | 0.84 (0.42 - 0.98) | 1.18 (1.03 - 1.40) |
| RBNW | 0.33 (0.48) | 0.82 (0.49 - 0.94) | 0.91 (0.59 - 0.98) | 1.04 (0.97 - 1.14) |
| RBSt | 0.47 (0.51) | 0.75 (0.43 - 0.92) | 0.93 (0.63 - 0.99) | 1.07 (1.01 - 1.17) |
| CABB | 0.05 (0.22) | 0.67 (0.47 - 0.83) | 0.85 (0.45 - 0.98) | 1.16 (1.04 - 1.31) |
| HLBB | 0.00 (0.00) | 0.68 (0.49 - 0.82) | 0.83 (0.41 - 0.97) | 1.14 (1.05 - 1.26) |
| TNTN | 0.04 (0.20) | 0.56 (0.32 - 0.77) | 0.81 (0.27 - 0.98) | 1.32 (1.23 - 1.41) |
| All | 0.22 (0.41) | 0.70 (0.44 - 0.86) | 0.87 (0.48 - 0.98) | 1.13 (0.97 - 1.43) |
| Sumatran tiger sand Sunda clouded leopards | | | | |
| RBNE | 0.30 (0.47) | 0.78 (0.01 - 0.99) | 0.61 (0.27 - 0.87) | 0.86 (0.50 - 1.31) |
| RBNW | 0.30 (0.47) | 1.00 (0.00 - 1.00) | 0.82 (0.49 - 0.96) | 0.92 (0.85 - 0.97) |
| RBSt | 0.06 (0.25) | 0.99 (0.00 - 1.00) | 0.40 (0.18 - 0.70) | 0.64 (0.22 - 0.87) |
| CABB | 0.00 (0.00) | 0.87 (0.00 - 1.00) | 0.76 (0.43 - 0.93) | 0.92 (0.72 - 1.11) |
| HLBB | 0.10 (0.31) | 0.86 (0.00 - 1.00) | 0.84 (0.52 - 0.96) | 0.99 (0.81 - 1.21) |
| TNTN | 0.00 (0.00) | 0.39 (0.01 - 0.95) | 0.86 (0.46 - 0.98) | 0.56 (0.22 - 2.12) |
| All | 0.13 (0.34) | 0.83 (0.00 - 0.99) | 0.70 (0.38 - 0.89) | 0.92 (0.45 - 1.40) |
| Sumatran tigers and dholes | | | | |
| RBNE | 0.20 (0.41) | 0.49 (0.00 - 1.00) | 0.94 (0.36 - 1.00) | 1.39 (0.89 - 3.77) |
| RBNW | 0.10 (0.31) | 0.02 (0.00 - 1.00) | 0.68 (0.25 - 0.92) | 1.31 (0.97 - 2.03) |
| RBSt | 0.09 (0.30) | 0.02 (0.00 - 1.00) | 0.47 (0.15 - 0.83) | 1.47 (1.03 - 2.51) |
| CABB | 0.00 (0.00) | 0.39 (0.00 - 1.00) | 0.92 (0.38 - 0.99) | 1.48 (1.02 - 3.01) |
| HLBB | 0.20 (0.41) | 0.44 (0.00 - 1.00) | 0.96 (0.41 - 1.00) | 1.44 (1.03 - 2.66) |
| TNTN | 0.00 (0.00) | 0.90 (0.00 - 1.00) | 0.97 (0.32 - 1.00) | 1.06 (0.96 - 1.19) |
| All | 0.10 (0.29) | 0.34 (0.00 - 1.00) | 0.79 (0.30 - 0.95) | 1.38 (0.47 - 5.78) |
