# Supplementary material for: Carnivores and their prey in Sumatra: Occupancy and activity in human-dominated forests
Source: PLoS One. 2022 Mar 18;17(3):e0265440. doi: 10.1371/journal.pone.0265440 (PMC8932565; doi:10.1371/journal.pone.0265440)
Supplement: S12 Table — (DOCX) [file pone.0265440.s013.docx]

**S13 Table. Spatial overlap between people (dominant, species A) and putative prey species (subordinate, species B) based on model-averaged ∆AICc ≤ 2 for 147 camera stations and across all study sites.** ψ^Ba^ is the probability of occupancy for species B, given species A is absent; ψ^BA^ is the probability of occupancy for species B, given species A is present; SIF is a species interaction factor where SIF = 1 indicates two species occurred independently of each other, an SIF >1 indicates overlap, whereas an SIF <1 suggests co-occurrence is less likely. A strong SIF is indicated by 95% CI not overlapping with 1; RBNE, Northeastern Bukit Rimbang Bukit Baling; RBNW, Northwestern Bukit Rimbang Bukit Baling; RBST, Southern Bukit Rimbang Bukit Baling; CABB, Bukit Bungkuk; HLBB, Bukit Betabuh; TNTN, Tesso Nilo; All, “All study sites”.

| **Study area** | **Naive spatially overlap (SD)** | **Mean PsiBa (95% CI)** | **Mean PsiBA (95% CI)** | **Mean SIF (95% CI)** |
| --- | --- | --- | --- | --- |
| People and Southern red muntjac | | | | |
| RBNE | 0.60 (0.50) | 0.98 (0.24 - 1.00) | 0.92 (0.79 - 0.97) | 0.98 (0.92 - 1.03) |
| RBNW | 0.50 (0.51) | 0.72 (0.27 - 0.93) | 0.72 (0.47 - 0.92) | 1.00 (0.55 - 1.80) |
| RBST | 0.47 (0.51) | 0.92 (0.34 - 0.99) | 0.91 (0.61 - 0.97) | 0.99 (0.89 - 1.09) |
| CABB | 0.10 (0.31) | 0.99 (0.26 - 1.00) | 0.91 (0.78 - 0.97) | 0.97 (0.94 - 1.00) |
| HLBB | 0.50 (0.51) | 0.99 (0.26 - 1.00) | 0.89 (0.74 - 0.96) | 0.96 (0.93 – 1.00) |
| TNTN | 0.84 (0.37) | 1.00 (0.17 - 1.00) | 0.93 (0.79 - 0.98) | 0.98 (0.98 - 0.99) |
| All | 0.51 (0.50) | 0.92 (0.26 - 0.98) | 0.87 (0.68 - 0.96) | 0.98 (0.79 - 1.17) |
| People and common wild pig | | | | |
| RBNE | 0.75 (0.44) | 0.96 (0.01 - 1.00) | 0.71 (0.57 - 0.82) | 0.93 (0.64 - 1.04) |
| RBNW | 0.13 (0.35) | 0.73 (0.01 - 0.99) | 0.18 (0.11 - 0.32) | 0.43 (-0.96 - 1.05) |
| RBST | 0.13 (0.34) | 0.88 (0.01 - 1.00) | 0.32 (0.21 - 0.48) | 0.63 (-0.36 - 0.96) |
| CABB | 0.00 (0.00) | 0.97 (0.01 - 1.00) | 0.66 (0.51 - 0.78) | 0.91 (0.70 - 1.00) |
| HLBB | 0.65 (0.49) | 0.97 (0.01 - 1.00) | 0.66 (0.51 - 0.79) | 0.91 (0.75 - 0.99) |
| TNTN | 0.64 (0.49) | 0.99 (0.00 - 1.00) | 0.92 (0.79 - 0.97) | 0.99 (0.99 - 0.99) |
| All | 0.35 (0.48) | 0.90 (0.01 - 1.00) | 0.54 (0.42 - 0.66) | 0.84 (-0.16 - 1.11) |
