# Supplementary material for: Carnivores and their prey in Sumatra: Occupancy and activity in human-dominated forests
Source: PLoS One. 2022 Mar 18;17(3):e0265440. doi: 10.1371/journal.pone.0265440 (PMC8932565; doi:10.1371/journal.pone.0265440)
Supplement: S13 Table — (DOCX) [file pone.0265440.s014.docx]

**S14 Table. Spatial overlap putative prey species (dominant, species A) and Sumatran tigers (subordinate, species B) model on model-averaged ∆AICc ≤ 2 for 147 camera stations across all study sites.** ψ^Ba^ is the probability of occupancy for species B, given species A is absent; ψ^BA^ is the probability of occupancy for species B, given species A is present; SIF is a species interaction factor where SIF = 1 indicates two species occurred independently of each other, an SIF >1 indicates overlap, whereas an SIF <1 suggests co-occurrence is less likely. A strong SIF is indicated by 95% CI not overlapping with 1; RBNE, Northeastern Bukit Rimbang Bukit Baling; RBNW, Northwestern Bukit Rimbang Bukit Baling; RBST, Southern Bukit Rimbang Bukit Baling; CABB, Bukit Bungkuk; HLBB, Bukit Betabuh; TNTN, Tesso Nilo; All, “All study sites”.

| **Study area** | **Naive spatially overlap (SD)** | **Mean PsiBa (95% CI)** | **Mean PsiBA (95% CI)** | **Mean SIF (95% CI)** |
| --- | --- | --- | --- | --- |
| Southern red muntjac and Sumatran tigers | | | | |
| RBNE | 0.25 (0.44) | 0.04 (0.00 - 1.00) | 0.61 (0.34 - 0.83) | 1.04 (0.93 - 1.19) |
| RBNW | 0.23 (0.43) | 0.40 (0.00 - 1.00) | 0.84 (0.59 - 0.94) | 1.16 (0.83 - 2.73) |
| RBST | 0.47 (0.51) | 0.10 (0.00 - 1.00) | 0.74 (0.52 - 0.88) | 1.11 (0.97 - 1.37) |
| CABB | 0.05 (0.22) | 0.00 (0.00 - 1.00) | 0.66 (0.41 - 0.85) | 1.03 (0.99 - 1.07) |
| HLBB | 0.10 (0.31) | 0.00 (0.00 - 1.00) | 0.67 (0.41 - 0.86) | 1.03 (1.00 - 1.07) |
| TNTN | 0.04 (0.20) | 0.00 (0.00 - 1.00) | 0.54 (0.21 - 0.84) | 1.01 (1.01 - 1.01) |
| All | 0.21 (0.41) | 0.11 (0.00 - 1.00) | 0.69 (0.43 - 0.87) | 1.08 (0.86 – 1.64) |
| Common wild pigs and Sumatran tigers | | | | |
| RBNE | 0.30 (0.47) | 0.21 (0.02 - 0.99) | 0.53 (0.36 - 0.71) | 1.14 (0.82 - 2.19) |
| RBNW | 0.07 (0.25) | 0.94 (0.03 - 1.00) | 0.23 (0.11 - 0.48) | 0.38 (-0.02 - 0.81) |
| RBST | 0.13 (0.34) | 0.80 (0.05 - 0.99) | 0.33 (0.18 - 0.53) | 0.63 (0.23 - 1.31) |
| CABB | 0.05 (0.22) | 0.29 (0.04 - 0.98) | 0.50 (0.34 - 0.66) | 1.09 (0.79 - 1.80) |
| HLBB | 0.15 (0.37) | 0.24 (0.02 - 0.98) | 0.50 (0.34 - 0.66) | 1.12 (0.85 - 1.67) |
| TNTN | 0.04 (0.20) | 0.00 (0.00 - 1.00) | 0.69 (0.44 - 0.86) | 1.10 (1.08 - 1.12) |
| All | 0.12 (0.32) | 0.47 (0.03 - 0.99) | 0.44 (0.28 - 0.64) | 0.98 (0.46 - 3.36) |
