# Supplementary material for: Carnivores and their prey in Sumatra: Occupancy and activity in human-dominated forests
Source: PLoS One. 2022 Mar 18;17(3):e0265440. doi: 10.1371/journal.pone.0265440 (PMC8932565; doi:10.1371/journal.pone.0265440)
Supplement: S14 Table — (DOCX) [file pone.0265440.s015.docx]

**S15 Table. Naive proportion of temporal patterns (diurnal, nocturnal and crepuscular) as indicated by the number of independent photographs, and the percentage (%) of all diel activity for people, large carnivores, and putative prey species across all study sites.** We assessed dominant activity patterns based on the percentage (%) of the activity period of each species based on the following three divisions of time: night–time/nocturnal (19h00 – 05h00), day–time/diurnal (07h00 – 17h00), and dawn/dusk/crepuscular (05h00 – 07h00 and 17h00 – 19h00) [1,2].

| **Species** | **Diurnal** | | **Nocturnal** | | **Crepuscular** | | **Total** | **Mardiana-watson-wheeler test (*W*)** |
| --- | --- | --- | --- | --- | --- | --- | --- | --- |
|  | **n** | **%** | **n** | **%** | **n** | **%** |  |  |
| **People** | 1561 | 85.86* | 56 | 3.08 | 201 | 11.06 | 1818 | *W* = 33.195, df = 8, *P* = 0.0001 |
| ***Large carnivores*** | | | | | | | | |
| **Cloudedleopard** | 59 | 31.05 | 88 | 46.32* | 43 | 22.63 | 190 | *W* = 11.57, df = 10, *P* = 0.3149 |
| **Dhole** | 32 | 86.49* | 1 | 2.7 | 4 | 10.81 | 37 | NA, value < 10 elements |
| **Malayansunbear** | 315 | 55.75* | 95 | 16.81 | 155 | 27.43 | 565 | *W* = 37.391, df = 10, *P* = 0.0000 |
| **Sumatrantiger** | 35 | 51.47* | 6 | 8.82 | 27 | 39.71 | 68 | *W* = 5.5621, df = 4, *P* = 0.2343 |
| ***Putative prey species*** | | | | | | | | |
| **Barkingdeer** | 403 | 61.25* | 63 | 9.57 | 192 | 29.18 | 658 | *W* = 5.6392, df = 10, *P* = 0.8446 |
| **Beardedpig** | 373 | 79.87* | 20 | 4.28 | 74 | 15.85 | 467 | *W* = 4.9487, df = 2, *P* = 0.0842 |
| **Mousedeer** | 89 | 23.86 | 128 | 34.32 | 156 | 41.82* | 373 | *W* = 69.683, df = 8, *P* = 0.0000 |
| **Sambardeer** | 3 | 20 | 8 | 53.33* | 4 | 26.67 | 15 | NA, value < 10 elements |
| **Sumatranserow** | 11 | 55* | 1 | 5 | 8 | 40 | 20 | NA, value < 10 elements |
| **Wildpig** | 445 | 72.71* | 61 | 9.97 | 106 | 17.32 | 612 | *W* = 17.589, df = 10, *P* = 0.0623 |

*the stronger domination with the highest percentage (%).

**Reference**

1. Azlan JMohd, Sharma DSK. The diversity and activity patterns of wild felids in a secondary forest in Peninsular Malaysia. Oryx. 2006;40: 36–41. doi:10.1017/S0030605306000147

2. Pusparini W, Wibisono HT, Reddy GV, Tarmizi T, Bharat P. Small and medium sized cats in Gunung Leuser National Park, Sumatra, Indonesia. CATnews Spec Issue 8 Spring 2014. 2014.
